# Supplementary material for: The Anticonvulsant Effects of Different Cannabis Extracts in a Zebrafish Model of Epilepsy
Source: Biomolecules. 2025 May 1;15(5):654. doi: 10.3390/biom15050654 (PMC12108886; doi:10.3390/biom15050654)
Supplement: Supplementary file 1 [file biomolecules-15-00654-s001.zip › biomolecules-3588986-supplementary.pdf]

Supplementary material

No PTZ control

ANOVA revealed a main Group effect for all strains (CAN-1: $F_{13,634} = 16.674$, $P < 0.001$; CAN-2: $F_{13,562} = 10.514$, $P < 0.001$; CAN-3: $F_{13,634} = 14.705$, $P < 0.001$).

Table S1. Descriptive Statistics

Test solution (µg/ml)	Test solution (µg/ml)	N	Mean	Std. Deviation	Std. Error	95% Confidence Interval for Mean		Minimum	Maximum
						Lower Bound	Upper Bound		
**CAN-1**	**E25 0.01**	36	3597.97	1756.50	292.75	3003.65	4192.29	582.85	7545.25
	**E25 0.1**	36	2489.69	2031.83	338.64	1802.22	3177.16	101.06	7300.84
	**E25 1.0**	36	3640.21	1567.10	261.18	3109.98	4170.44	898.72	7030.92
	**E25 10**	36	5014.33	1518.24	253.04	4500.63	5528.03	1928.22	8890.20
	**E100 0.01**	36	3235.37	2003.91	333.98	2557.34	3913.39	588.27	8195.02
	**E100 0.1**	36	2387.09	1742.11	290.35	1797.64	2976.53	176.15	7331.32
	**E100 1.0**	36	2106.88	1639.27	273.21	1552.23	2661.53	286.57	8402.18
	**E100 10**	36	5227.75	1233.07	205.51	4810.54	5644.97	2497.15	7848.89
	**EA 0.01**	36	2371.60	1923.95	320.66	1720.63	3022.57	0.00	7165.74
	**EA 0.1**	36	2539.66	1759.61	293.27	1944.30	3135.03	264.99	6616.99
	**EA 1.0**	36	2545.92	1663.02	277.17	1983.24	3108.60	319.25	6271.88
	**EA 10**	36	3640.87	1725.84	287.64	3056.93	4224.81	58.56	7429.15
	**DMSO**	108	2405.61	1606.65	154.60	2099.13	2712.08	0.19	6538.32
	**VPA**	108	1805.41	1706.92	164.25	1479.80	2131.01	0.00	8031.48
**CAN-2**	**E25 0.01**	24	3529.18	1931.53	394.27	2713.56	4344.79	867.42	8054.91
	**E25 0.1**	24	5229.42	2004.19	409.10	4383.12	6075.71	1324.27	8240.90
	**E25 1.0**	24	3801.58	1407.96	287.40	3207.05	4396.11	852.94	6502.29
	**E25 10**	24	5087.50	1644.34	335.65	4393.16	5781.85	2744.26	10037.05
	**E100 0.01**	36	3603.17	1826.43	304.41	2985.20	4221.15	1.68	8645.72
	**E100 0.1**	36	3357.79	1496.25	249.38	2851.53	3864.05	910.24	6926.48
	**E100 1.0**	36	4747.16	1868.89	311.48	4114.82	5379.50	1862.77	8736.84
	**E100 10**	36	3324.73	597.73	99.62	3122.49	3526.97	1838.23	4503.21
	**EA 0.01**	36	3946.30	2073.20	345.53	3244.83	4647.77	9.31	8119.74
	**EA 0.1**	36	4139.16	1741.58	290.26	3549.90	4728.43	482.36	8401.55
	**EA 1.0**	36	3793.93	1847.68	307.95	3168.77	4419.10	337.59	8004.81
	**EA 10**	36	5228.60	1319.61	219.94	4782.11	5675.09	2455.40	7805.05
	**DMSO**	96	3473.60	1821.37	185.89	3104.56	3842.65	5.14	9649.62
	**VPA**	96	2436.22	1753.13	178.93	2081.00	2791.44	0.00	6748.17
**CAN-3**	**E25 0.01**	36	3778.25	1688.99	281.50	3206.78	4349.72	1222.69	8133.80
	**E25 0.1**	36	3716.09	2057.07	342.84	3020.08	4412.10	0.00	9209.84
	**E25 1.0**	36	3835.21	1948.03	324.67	3176.09	4494.33	783.42	8651.99
	**E25 10**	36	5945.78	1243.27	207.21	5525.12	6366.44	3593.66	9036.49
	**E100 0.01**	36	3794.81	1831.08	305.18	3175.26	4414.36	1058.06	7784.40
	**E100 0.1**	36	4273.23	2333.11	388.85	3483.82	5062.64	346.35	11097.83
	**E100 1.0**	36	5771.74	2390.77	398.46	4962.81	6580.66	160.20	10110.17
	**E100 10**	36	3321.01	791.24	131.87	3053.29	3588.73	1530.53	5564.93
	**EA 0.01**	36	3166.17	1897.12	316.19	2524.28	3808.06	16.50	7147.86

**EA 0.1**	36	3222.14	1810.55	301.76	2609.54	3834.74	489.06	8493.92
**EA 1.0**	36	2577.40	1339.38	223.23	2124.22	3030.58	980.94	5843.40
**EA 10**	36	5189.19	917.47	152.91	4878.76	5499.62	3517.52	7625.38
**DMSO**	108	3954.12	1698.69	163.46	3630.09	4278.16	824.24	8356.88
**VPA**	108	2638.91	2052.46	197.50	2247.40	3030.43	0.00	7783.78

Table S2. Effect Sizes

		Point Estimate	95% Confidence Interval Lower	Upper
**CAN-1**	**Eta-squared**	0.255	0.185	0.295
	**Epsilon-squared**	0.240	0.169	0.280
	**Omega-squared Fixed-effect**	0.239	0.168	0.280
	**Omega-squared Random-effect**	0.024	0.015	0.029
**CAN-2**	**Eta-squared**	0.196	0.124	0.235
	**Epsilon-squared**	0.177	0.104	0.217
	**Omega-squared Fixed-effect**	0.177	0.103	0.217
	**Omega-squared Random-effect**	0.016	0.009	0.021
**CAN-3**	**Eta-squared**	0.232	0.163	0.271
	**Epsilon-squared**	0.216	0.146	0.256
	**Omega-squared Fixed-effect**	0.216	0.146	0.256
	**Omega-squared Random-effect**	0.021	0.013	0.026

Table S3. Post-hoc tests

Test solution (µg/ml)	Test solution (µg/ml)	Mean Difference	Std. Error	Sig.	95% Confidence Interval Lower Bound	Upper Bound
CAN-1						
**E25 0.01**	**E25 0.1**	1108.27993	401.54686	0.248	-243.6520	2460.2119
	**E25 1.0**	-42.23808	401.54686	1.000	-1394.1700	1309.6939
	**E25 10**	-1416.35631*	401.54686	0.030	-2768.2883	-64.4244
	**VPA**	1792.56286*	327.86164	0.000	688.7151	2896.4107
	**DMSO**	1192.36282*	327.86164	0.021	88.5150	2296.2106
	**E100 0.01**	362.60133	401.54686	1.000	-989.3306	1714.5333
	**E100 0.1**	1210.88157	401.54686	0.135	-141.0504	2562.8135
	**E100 1.0**	1491.09291*	401.54686	0.016	139.1610	2843.0249
	**E100 10**	-1629.78425*	401.54686	0.004	-2981.7162	-277.8523
	**EA 0.01**	1226.37100	401.54686	0.122	-125.5610	2578.3029
	**EA 0.1**	1058.30766	401.54686	0.320	-293.6243	2410.2396
	**EA 1.0**	1052.04879	401.54686	0.330	-299.8832	2403.9807
	**EA 10**	-42.89604	401.54686	1.000	-1394.8280	1309.0359
**E25 0.1**	**E25 0.01**	-1108.27993	401.54686	0.248	-2460.2119	243.6520
	**E25 1.0**	-1150.51801	401.54686	0.196	-2502.4500	201.4139
	**E25 10**	-2524.63624*	401.54686	0.000	-3876.5682	-1172.7043
	**VPA**	684.28293	327.86164	0.710	-419.5649	1788.1307
	**DMSO**	84.08289	327.86164	1.000	-1019.7649	1187.9307
	**E100 0.01**	-745.67860	401.54686	0.849	-2097.6106	606.2533
	**E100 0.1**	102.60164	401.54686	1.000	-1249.3303	1454.5336
	**E100 1.0**	382.81298	401.54686	1.000	-969.1190	1734.7449
	**E100 10**	-2738.06418*	401.54686	0.000	-4089.9961	-1386.1322
	**EA 0.01**	118.09107	401.54686	1.000	-1233.8409	1470.0230
	**EA 0.1**	-49.97227	401.54686	1.000	-1401.9042	1301.9597
	**EA 1.0**	-56.23114	401.54686	1.000	-1408.1631	1295.7008
	**EA 10**	-1151.17597	401.54686	0.195	-2503.1079	200.7560
**E25 1.0**	**E25 0.01**	42.23808	401.54686	1.000	-1309.6939	1394.1700
	**E25 0.1**	1150.51801	401.54686	0.196	-201.4139	2502.4500
	**E25 10**	-1374.11823*	401.54686	0.042	-2726.0502	-22.1863
	**VPA**	1834.80095*	327.86164	0.000	730.9531	2938.6488
	**DMSO**	1234.60090*	327.86164	0.013	130.7531	2338.4487
	**E100 0.01**	404.83941	401.54686	0.999	-947.0925	1756.7714
	**E100 0.1**	1253.11965	401.54686	0.102	-98.8123	2605.0516
	**E100 1.0**	1533.33100*	401.54686	0.011	181.3990	2885.2629

	**E100 10**	-1587.54616*	401.54686	0.007	-2939.4781	-235.6142
	**EA 0.01**	1268.60908	401.54686	0.092	-83.3229	2620.5410
	**EA 0.1**	1100.54575	401.54686	0.258	-251.3862	2452.4777
	**EA 1.0**	1094.28687	401.54686	0.267	-257.6451	2446.2188
	**EA 10**	-0.65795	401.54686	1.000	-1352.5899	1351.2740
**E25 10**	**E25 0.01**	1416.35631*	401.54686	0.030	64.4244	2768.2883
	**E25 0.1**	2524.63624*	401.54686	0.000	1172.7043	3876.5682
	**E25 1.0**	1374.11823*	401.54686	0.042	22.1863	2726.0502
	**VPA**	3208.91918*	327.86164	0.000	2105.0714	4312.7670
	**DMSO**	2608.71913*	327.86164	0.000	1504.8713	3712.5669
	**E100 0.01**	1778.95764*	401.54686	0.001	427.0257	3130.8896
	**E100 0.1**	2627.23788*	401.54686	0.000	1275.3059	3979.1698
	**E100 1.0**	2907.44923*	401.54686	0.000	1555.5173	4259.3812
	**E100 10**	-213.42793	401.54686	1.000	-1565.3599	1138.5040
	**EA 0.01**	2642.72731*	401.54686	0.000	1290.7954	3994.6593
	**EA 0.1**	2474.66398*	401.54686	0.000	1122.7320	3826.5959
	**EA 1.0**	2468.40510*	401.54686	0.000	1116.4732	3820.3371
	**EA 10**	1373.46028*	401.54686	0.042	21.5283	2725.3922
**VPA**	**E25 0.01**	-1792.56286*	327.86164	0.000	-2896.4107	-688.7151
	**E25 0.1**	-684.28293	327.86164	0.710	-1788.1307	419.5649
	**E25 1.0**	-1834.80095*	327.86164	0.000	-2938.6488	-730.9531
	**E25 10**	-3208.91918*	327.86164	0.000	-4312.7670	-2105.0714
	**DMSO**	-600.20005	231.83319	0.350	-1380.7383	180.3382
	**E100 0.01**	-1429.96154*	327.86164	0.001	-2533.8093	-326.1137
	**E100 0.1**	-581.68129	327.86164	0.887	-1685.5291	522.1665
	**E100 1.0**	-301.46995	327.86164	1.000	-1405.3178	802.3779
	**E100 10**	-3422.34711*	327.86164	0.000	-4526.1949	-2318.4993
	**EA 0.01**	-566.19187	327.86164	0.906	-1670.0397	537.6559
	**EA 0.1**	-734.25520	327.86164	0.601	-1838.1030	369.5926
	**EA 1.0**	-740.51408	327.86164	0.586	-1844.3619	363.3337
	**EA 10**	-1835.45890*	327.86164	0.000	-2939.3067	-731.6111
**DMSO**	**E25 0.01**	-1192.36282*	327.86164	0.021	-2296.2106	-88.5150
	**E25 0.1**	-84.08289	327.86164	1.000	-1187.9307	1019.7649
	**E25 1.0**	-1234.60090*	327.86164	0.013	-2338.4487	-130.7531
	**E25 10**	-2608.71913*	327.86164	0.000	-3712.5669	-1504.8713
	**VPA**	600.20005	231.83319	0.350	-180.3382	1380.7383
	**E100 0.01**	-829.76149	327.86164	0.388	-1933.6093	274.0863
	**E100 0.1**	18.51875	327.86164	1.000	-1085.3291	1122.3666
	**E100 1.0**	298.73009	327.86164	1.000	-805.1177	1402.5779
	**E100 10**	-2822.14706*	327.86164	0.000	-3925.9949	-1718.2992
	**EA 0.01**	34.00818	327.86164	1.000	-1069.8396	1137.8560
	**EA 0.1**	-134.05515	327.86164	1.000	-1237.9030	969.7927
	**EA 1.0**	-140.31403	327.86164	1.000	-1244.1618	963.5338
	**EA 10**	-1235.25886*	327.86164	0.013	-2339.1067	-131.4110
**E100 0.01**	**E25 0.01**	-362.60133	401.54686	1.000	-1714.5333	989.3306
	**E25 0.1**	745.67860	401.54686	0.849	-606.2533	2097.6106
	**E25 1.0**	-404.83941	401.54686	0.999	-1756.7714	947.0925
	**E25 10**	-1778.95764*	401.54686	0.001	-3130.8896	-427.0257
	**VPA**	1429.96154*	327.86164	0.001	326.1137	2533.8093
	**DMSO**	829.76149	327.86164	0.388	-274.0863	1933.6093
	**E100 0.1**	848.28024	401.54686	0.693	-503.6517	2200.2122
	**E100 1.0**	1128.49158	401.54686	0.222	-223.4404	2480.4235
	**E100 10**	-1992.38558*	401.54686	0.000	-3344.3175	-640.4536
	**EA 0.01**	863.76967	401.54686	0.665	-488.1623	2215.7016
	**EA 0.1**	695.70633	401.54686	0.904	-656.2256	2047.6383
	**EA 1.0**	689.44746	401.54686	0.910	-662.4845	2041.3794
	**EA 10**	-405.49737	401.54686	0.999	-1757.4293	946.4346
**E100 0.1**	**E25 0.01**	-1210.88157	401.54686	0.135	-2562.8135	141.0504
	**E25 0.1**	-102.60164	401.54686	1.000	-1454.5336	1249.3303
	**E25 1.0**	-1253.11965	401.54686	0.102	-2605.0516	98.8123
	**E25 10**	-2627.23788*	401.54686	0.000	-3979.1698	-1275.3059

	**VPA**	581.68129	327.86164	0.887	-522.1665	1685.5291
	**DMSO**	-18.51875	327.86164	1.000	-1122.3666	1085.3291
	**E100 0.01**	-848.28024	401.54686	0.693	-2200.2122	503.6517
	**E100 1.0**	280.21134	401.54686	1.000	-1071.7206	1632.1433
	**E100 10**	-2840.66582*	401.54686	0.000	-4192.5978	-1488.7339
	**EA 0.01**	15.48943	401.54686	1.000	-1336.4425	1367.4214
	**EA 0.1**	-152.57391	401.54686	1.000	-1504.5059	1199.3580
	**EA 1.0**	-158.83278	401.54686	1.000	-1510.7647	1193.0992
	**EA 10**	-1253.77761	401.54686	0.102	-2605.7096	98.1543
**E100 1.0**	**E25 0.01**	-1491.09291*	401.54686	0.016	-2843.0249	-139.1610
	**E25 0.1**	-382.81298	401.54686	1.000	-1734.7449	969.1190
	**E25 1.0**	-1533.33100*	401.54686	0.011	-2885.2629	-181.3990
	**E25 10**	-2907.44923*	401.54686	0.000	-4259.3812	-1555.5173
	**VPA**	301.46995	327.86164	1.000	-802.3779	1405.3178
	**DMSO**	-298.73009	327.86164	1.000	-1402.5779	805.1177
	**E100 0.01**	-1128.49158	401.54686	0.222	-2480.4235	223.4404
	**E100 0.1**	-280.21134	401.54686	1.000	-1632.1433	1071.7206
	**E100 10**	-3120.87716*	401.54686	0.000	-4472.8091	-1768.9452
	**EA 0.01**	-264.72191	401.54686	1.000	-1616.6539	1087.2100
	**EA 0.1**	-432.78525	401.54686	0.998	-1784.7172	919.1467
	**EA 1.0**	-439.04412	401.54686	0.998	-1790.9761	912.8878
	**EA 10**	-1533.98895*	401.54686	0.011	-2885.9209	-182.0570
**E100 10**	**E25 0.01**	1629.78425*	401.54686	0.004	277.8523	2981.7162
	**E25 0.1**	2738.06418*	401.54686	0.000	1386.1322	4089.9961
	**E25 1.0**	1587.54616*	401.54686	0.007	235.6142	2939.4781
	**E25 10**	213.42793	401.54686	1.000	-1138.5040	1565.3599
	**VPA**	3422.34711*	327.86164	0.000	2318.4993	4526.1949
	**DMSO**	2822.14706*	327.86164	0.000	1718.2992	3925.9949
	**E100 0.01**	1992.38558*	401.54686	0.000	640.4536	3344.3175
	**E100 0.1**	2840.66582*	401.54686	0.000	1488.7339	4192.5978
	**E100 1.0**	3120.87716*	401.54686	0.000	1768.9452	4472.8091
	**EA 0.01**	2856.15525*	401.54686	0.000	1504.2233	4208.0872
	**EA 0.1**	2688.09191*	401.54686	0.000	1336.1600	4040.0239
	**EA 1.0**	2681.83303*	401.54686	0.000	1329.9011	4033.7650
	**EA 10**	1586.88821*	401.54686	0.007	234.9563	2938.8202
**EA 0.01**	**E25 0.01**	-1226.37100	401.54686	0.122	-2578.3029	125.5610
	**E25 0.1**	-118.09107	401.54686	1.000	-1470.0230	1233.8409
	**E25 1.0**	-1268.60908	401.54686	0.092	-2620.5410	83.3229
	**E25 10**	-2642.72731*	401.54686	0.000	-3994.6593	-1290.7954
	**VPA**	566.19187	327.86164	0.906	-537.6559	1670.0397
	**DMSO**	-34.00818	327.86164	1.000	-1137.8560	1069.8396
	**E100 0.01**	-863.76967	401.54686	0.665	-2215.7016	488.1623
	**E100 0.1**	-15.48943	401.54686	1.000	-1367.4214	1336.4425
	**E100 1.0**	264.72191	401.54686	1.000	-1087.2100	1616.6539
	**E100 10**	-2856.15525*	401.54686	0.000	-4208.0872	-1504.2233
	**EA 0.1**	-168.06333	401.54686	1.000	-1519.9953	1183.8686
	**EA 1.0**	-174.32221	401.54686	1.000	-1526.2542	1177.6097
	**EA 10**	-1269.26704	401.54686	0.092	-2621.1990	82.6649
**EA 0.1**	**E25 0.01**	-1058.30766	401.54686	0.320	-2410.2396	293.6243
	**E25 0.1**	49.97227	401.54686	1.000	-1301.9597	1401.9042
	**E25 1.0**	-1100.54575	401.54686	0.258	-2452.4777	251.3862
	**E25 10**	-2474.66398*	401.54686	0.000	-3826.5959	-1122.7320
	**VPA**	734.25520	327.86164	0.601	-369.5926	1838.1030
	**DMSO**	134.05515	327.86164	1.000	-969.7927	1237.9030
	**E100 0.01**	-695.70633	401.54686	0.904	-2047.6383	656.2256
	**E100 0.1**	152.57391	401.54686	1.000	-1199.3580	1504.5059
	**E100 1.0**	432.78525	401.54686	0.998	-919.1467	1784.7172
	**E100 10**	-2688.09191*	401.54686	0.000	-4040.0239	-1336.1600
	**EA 0.01**	168.06333	401.54686	1.000	-1183.8686	1519.9953
	**EA 1.0**	-6.25888	401.54686	1.000	-1358.1908	1345.6731
	**EA 10**	-1101.20370	401.54686	0.257	-2453.1357	250.7282
**EA 1.0**	**E25 0.01**	-1052.04879	401.54686	0.330	-2403.9807	299.8832
	**E25 0.1**	56.23114	401.54686	1.000	-1295.7008	1408.1631

	**E25 1.0**	-1094.28687	401.54686	0.267	-2446.2188	257.6451
	**E25 10**	-2468.40510*	401.54686	0.000	-3820.3371	-1116.4732
	**VPA**	740.51408	327.86164	0.586	-363.3337	1844.3619
	**DMSO**	140.31403	327.86164	1.000	-963.5338	1244.1618
	**E100 0.01**	-689.44746	401.54686	0.910	-2041.3794	662.4845
	**E100 0.1**	158.83278	401.54686	1.000	-1193.0992	1510.7647
	**E100 1.0**	439.04412	401.54686	0.998	-912.8878	1790.9761
	**E100 10**	-2681.83303*	401.54686	0.000	-4033.7650	-1329.9011
	**EA 0.01**	174.32221	401.54686	1.000	-1177.6097	1526.2542
	**EA 0.1**	6.25888	401.54686	1.000	-1345.6731	1358.1908
	**EA 10**	-1094.94483	401.54686	0.266	-2446.8768	256.9871
**EA 10**	**E25 0.01**	42.89604	401.54686	1.000	-1309.0359	1394.8280
	**E25 0.1**	1151.17597	401.54686	0.195	-200.7560	2503.1079
	**E25 1.0**	0.65795	401.54686	1.000	-1351.2740	1352.5899
	**E25 10**	-1373.46028*	401.54686	0.042	-2725.3922	-21.5283
	**VPA**	1835.45890*	327.86164	0.000	731.6111	2939.3067
	**DMSO**	1235.25886*	327.86164	0.013	131.4110	2339.1067
	**E100 0.01**	405.49737	401.54686	0.999	-946.4346	1757.4293
	**E100 0.1**	1253.77761	401.54686	0.102	-98.1543	2605.7096
	**E100 1.0**	1533.98895*	401.54686	0.011	182.0570	2885.9209
	**E100 10**	-1586.88821*	401.54686	0.007	-2938.8202	-234.9563
	**EA 0.01**	1269.26704	401.54686	0.092	-82.6649	2621.1990
	**EA 0.1**	1101.20370	401.54686	0.257	-250.7282	2453.1357
	**EA 1.0**	1094.94483	401.54686	0.266	-256.9871	2446.8768
**CAN-2**						
**E25 0.01**	**E25 0.1**	-1700.24159*	496.12995	0.042	-3371.4565	-29.0267
	**E25 1.0**	-272.40238	496.12995	1.000	-1943.6173	1398.8125
	**E25 10**	-1558.32550	496.12995	0.097	-3229.5404	112.8894
	**VPA**	1092.95964	392.22517	0.234	-228.2518	2414.1710
	**DMSO**	55.57388	392.22517	1.000	-1265.6375	1376.7853
	**E100 0.01**	-73.99485	452.90261	1.000	-1599.5984	1451.6087
	**E100 0.1**	171.38815	452.90261	1.000	-1354.2154	1696.9917
	**E100 1.0**	-1217.97989	452.90261	0.288	-2743.5834	307.6236
	**E100 10**	204.44967	452.90261	1.000	-1321.1538	1730.0532
	**EA 0.01**	-417.12428	452.90261	1.000	-1942.7278	1108.4792
	**EA 0.1**	-609.98585	452.90261	0.987	-2135.5894	915.6177
	**EA 1.0**	-264.75685	452.90261	1.000	-1790.3604	1260.8467
	**EA 10**	-1699.42132*	452.90261	0.014	-3225.0248	-173.8178
**E25 0.1**	**E25 0.01**	1700.24159*	496.12995	0.042	29.0267	3371.4565
	**E25 1.0**	1427.83920	496.12995	0.190	-243.3757	3099.0541
	**E25 10**	141.91608	496.12995	1.000	-1529.2988	1813.1310
	**VPA**	2793.20123*	392.22517	0.000	1471.9898	4114.4126
	**DMSO**	1755.81546*	392.22517	0.001	434.6041	3077.0269
	**E100 0.01**	1626.24673*	452.90261	0.024	100.6432	3151.8502
	**E100 0.1**	1871.62973*	452.90261	0.003	346.0262	3397.2332
	**E100 1.0**	482.26169	452.90261	0.999	-1043.3418	2007.8652
	**E100 10**	1904.69126*	452.90261	0.002	379.0877	3430.2948
	**EA 0.01**	1283.11731	452.90261	0.211	-242.4862	2808.7208
	**EA 0.1**	1090.25573	452.90261	0.476	-435.3478	2615.8592
	**EA 1.0**	1435.48474	452.90261	0.090	-90.1188	2961.0883
	**EA 10**	0.82026	452.90261	1.000	-1524.7833	1526.4238
**E25 1.0**	**E25 0.01**	272.40238	496.12995	1.000	-1398.8125	1943.6173
	**E25 0.1**	-1427.83920	496.12995	0.190	-3099.0541	243.3757
	**E25 10**	-1285.92312	496.12995	0.348	-2957.1380	385.2918
	**VPA**	1365.36202*	392.22517	0.035	44.1506	2686.5734
	**DMSO**	327.97626	392.22517	1.000	-993.2351	1649.1877
	**E100 0.01**	198.40753	452.90261	1.000	-1327.1960	1724.0110
	**E100 0.1**	443.79053	452.90261	0.999	-1081.8130	1969.3940
	**E100 1.0**	-945.57751	452.90261	0.710	-2471.1810	580.0260
	**E100 10**	476.85205	452.90261	0.999	-1048.7515	2002.4556
	**EA 0.01**	-144.72189	452.90261	1.000	-1670.3254	1380.8816
	**EA 0.1**	-337.58347	452.90261	1.000	-1863.1870	1188.0200
	**EA 1.0**	7.64554	452.90261	1.000	-1517.9580	1533.2491
	**EA 10**	-1427.01894	452.90261	0.095	-2952.6225	98.5846

**E25 10**	**E25 0.01**	1558.32550	496.12995	0.097	-112.8894	3229.5404
	**E25 0.1**	-141.91608	496.12995	1.000	-1813.1310	1529.2988
	**E25 1.0**	1285.92312	496.12995	0.348	-385.2918	2957.1380
	**VPA**	2651.28515*	392.22517	0.000	1330.0737	3972.4965
	**DMSO**	1613.89938*	392.22517	0.004	292.6880	2935.1108
	**E100 0.01**	1484.33065	452.90261	0.066	-41.2729	3009.9342
	**E100 0.1**	1729.71365*	452.90261	0.011	204.1101	3255.3172
	**E100 1.0**	340.34561	452.90261	1.000	-1185.2579	1865.9491
	**E100 10**	1762.77517*	452.90261	0.008	237.1717	3288.3787
	**EA 0.01**	1141.20123	452.90261	0.396	-384.4023	2666.8047
	**EA 0.1**	948.33965	452.90261	0.705	-577.2639	2473.9432
	**EA 1.0**	1293.56866	452.90261	0.200	-232.0349	2819.1722
	**EA 10**	-141.09582	452.90261	1.000	-1666.6993	1384.5077
**VPA**	**E25 0.01**	-1092.95964	392.22517	0.234	-2414.1710	228.2518
	**E25 0.1**	-2793.20123*	392.22517	0.000	-4114.4126	-1471.9898
	**E25 1.0**	-1365.36202*	392.22517	0.035	-2686.5734	-44.1506
	**E25 10**	-2651.28515*	392.22517	0.000	-3972.4965	-1330.0737
	**DMSO**	-1037.38576*	248.06498	0.003	-1872.9932	-201.7783
	**E100 0.01**	-1166.95449*	335.88157	0.036	-2298.3723	-35.5366
	**E100 0.1**	-921.57149	335.88157	0.257	-2052.9893	209.8464
	**E100 1.0**	-2310.93953*	335.88157	0.000	-3442.3574	-1179.5217
	**E100 10**	-888.50997	335.88157	0.314	-2019.9278	242.9079
	**EA 0.01**	-1510.08392*	335.88157	0.001	-2641.5018	-378.6661
	**EA 0.1**	-1702.94549*	335.88157	0.000	-2834.3633	-571.5276
	**EA 1.0**	-1357.71649*	335.88157	0.005	-2489.1343	-226.2986
	**EA 10**	-2792.38097*	335.88157	0.000	-3923.7988	-1660.9631
**DMSO**	**E25 0.01**	-55.57388	392.22517	1.000	-1376.7853	1265.6375
	**E25 0.1**	-1755.81546*	392.22517	0.001	-3077.0269	-434.6041
	**E25 1.0**	-327.97626	392.22517	1.000	-1649.1877	993.2351
	**E25 10**	-1613.89938*	392.22517	0.004	-2935.1108	-292.6880
	**VPA**	1037.38576*	248.06498	0.003	201.7783	1872.9932
	**E100 0.01**	-129.56873	335.88157	1.000	-1260.9866	1001.8491
	**E100 0.1**	115.81427	335.88157	1.000	-1015.6036	1247.2321
	**E100 1.0**	-1273.55377*	335.88157	0.012	-2404.9716	-142.1359
	**E100 10**	148.87579	335.88157	1.000	-982.5421	1280.2936
	**EA 0.01**	-472.69815	335.88157	0.981	-1604.1160	658.7197
	**EA 0.1**	-665.55973	335.88157	0.779	-1796.9776	465.8581
	**EA 1.0**	-320.33072	335.88157	1.000	-1451.7486	811.0871
	**EA 10**	-1754.99520*	335.88157	0.000	-2886.4130	-623.5774
**E100 0.01**	**E25 0.01**	73.99485	452.90261	1.000	-1451.6087	1599.5984
	**E25 0.1**	-1626.24673*	452.90261	0.024	-3151.8502	-100.6432
	**E25 1.0**	-198.40753	452.90261	1.000	-1724.0110	1327.1960
	**E25 10**	-1484.33065	452.90261	0.066	-3009.9342	41.2729
	**VPA**	1166.95449*	335.88157	0.036	35.5366	2298.3723
	**DMSO**	129.56873	335.88157	1.000	-1001.8491	1260.9866
	**E100 0.1**	245.38300	405.08841	1.000	-1119.1583	1609.9243
	**E100 1.0**	-1143.98504	405.08841	0.216	-2508.5263	220.5562
	**E100 10**	278.44452	405.08841	1.000	-1086.0967	1642.9858
	**EA 0.01**	-343.12942	405.08841	1.000	-1707.6707	1021.4118
	**EA 0.1**	-535.99100	405.08841	0.989	-1900.5323	828.5503
	**EA 1.0**	-190.76199	405.08841	1.000	-1555.3033	1173.7793
	**EA 10**	-1625.42647*	405.08841	0.005	-2989.9677	-260.8852
**E100 0.1**	**E25 0.01**	-171.38815	452.90261	1.000	-1696.9917	1354.2154
	**E25 0.1**	-1871.62973*	452.90261	0.003	-3397.2332	-346.0262
	**E25 1.0**	-443.79053	452.90261	0.999	-1969.3940	1081.8130
	**E25 10**	-1729.71365*	452.90261	0.011	-3255.3172	-204.1101
	**VPA**	921.57149	335.88157	0.257	-209.8464	2052.9893
	**DMSO**	-115.81427	335.88157	1.000	-1247.2321	1015.6036
	**E100 0.01**	-245.38300	405.08841	1.000	-1609.9243	1119.1583
	**E100 1.0**	-1389.36804*	405.08841	0.041	-2753.9093	-24.8268
	**E100 10**	33.06152	405.08841	1.000	-1331.4797	1397.6028
	**EA 0.01**	-588.51242	405.08841	0.975	-1953.0537	776.0288

	**EA 0.1**	-781.37400	405.08841	0.810	-2145.9153	583.1673
	**EA 1.0**	-436.14499	405.08841	0.999	-1800.6863	928.3963
	**EA 10**	-1870.80947*	405.08841	0.000	-3235.3507	-506.2682
**E100 1.0**	**E25 0.01**	1217.97989	452.90261	0.288	-307.6236	2743.5834
	**E25 0.1**	-482.26169	452.90261	0.999	-2007.8652	1043.3418
	**E25 1.0**	945.57751	452.90261	0.710	-580.0260	2471.1810
	**E25 10**	-340.34561	452.90261	1.000	-1865.9491	1185.2579
	**VPA**	2310.93953*	335.88157	0.000	1179.5217	3442.3574
	**DMSO**	1273.55377*	335.88157	0.012	142.1359	2404.9716
	**E100 0.01**	1143.98504	405.08841	0.216	-220.5562	2508.5263
	**E100 0.1**	1389.36804*	405.08841	0.041	24.8268	2753.9093
	**E100 10**	1422.42956*	405.08841	0.032	57.8883	2786.9708
	**EA 0.01**	800.85562	405.08841	0.782	-563.6856	2165.3969
	**EA 0.1**	607.99404	405.08841	0.967	-756.5472	1972.5353
	**EA 1.0**	953.22305	405.08841	0.516	-411.3182	2317.7643
	**EA 10**	-481.44143	405.08841	0.996	-1845.9827	883.0998
**E100 10**	**E25 0.01**	-204.44967	452.90261	1.000	-1730.0532	1321.1538
	**E25 0.1**	-1904.69126*	452.90261	0.002	-3430.2948	-379.0877
	**E25 1.0**	-476.85205	452.90261	0.999	-2002.4556	1048.7515
	**E25 10**	-1762.77517*	452.90261	0.008	-3288.3787	-237.1717
	**VPA**	888.50997	335.88157	0.314	-242.9079	2019.9278
	**DMSO**	-148.87579	335.88157	1.000	-1280.2936	982.5421
	**E100 0.01**	-278.44452	405.08841	1.000	-1642.9858	1086.0967
	**E100 0.1**	-33.06152	405.08841	1.000	-1397.6028	1331.4797
	**E100 1.0**	-1422.42956*	405.08841	0.032	-2786.9708	-57.8883
	**EA 0.01**	-621.57395	405.08841	0.961	-1986.1152	742.9673
	**EA 0.1**	-814.43552	405.08841	0.761	-2178.9768	550.1057
	**EA 1.0**	-469.20652	405.08841	0.997	-1833.7478	895.3347
	**EA 10**	-1903.87099*	405.08841	0.000	-3268.4123	-539.3297
**EA 0.01**	**E25 0.01**	417.12428	452.90261	1.000	-1108.4792	1942.7278
	**E25 0.1**	-1283.11731	452.90261	0.211	-2808.7208	242.4862
	**E25 1.0**	144.72189	452.90261	1.000	-1380.8816	1670.3254
	**E25 10**	-1141.20123	452.90261	0.396	-2666.8047	384.4023
	**VPA**	1510.08392*	335.88157	0.001	378.6661	2641.5018
	**DMSO**	472.69815	335.88157	0.981	-658.7197	1604.1160
	**E100 0.01**	343.12942	405.08841	1.000	-1021.4118	1707.6707
	**E100 0.1**	588.51242	405.08841	0.975	-776.0288	1953.0537
	**E100 1.0**	-800.85562	405.08841	0.782	-2165.3969	563.6856
	**E100 10**	621.57395	405.08841	0.961	-742.9673	1986.1152
	**EA 0.1**	-192.86158	405.08841	1.000	-1557.4028	1171.6797
	**EA 1.0**	152.36743	405.08841	1.000	-1212.1738	1516.9087
	**EA 10**	-1282.29705	405.08841	0.091	-2646.8383	82.2442
**EA 0.1**	**E25 0.01**	609.98585	452.90261	0.987	-915.6177	2135.5894
	**E25 0.1**	-1090.25573	452.90261	0.476	-2615.8592	435.3478
	**E25 1.0**	337.58347	452.90261	1.000	-1188.0200	1863.1870
	**E25 10**	-948.33965	452.90261	0.705	-2473.9432	577.2639
	**VPA**	1702.94549*	335.88157	0.000	571.5276	2834.3633
	**DMSO**	665.55973	335.88157	0.779	-465.8581	1796.9776
	**E100 0.01**	535.99100	405.08841	0.989	-828.5503	1900.5323
	**E100 0.1**	781.37400	405.08841	0.810	-583.1673	2145.9153
	**E100 1.0**	-607.99404	405.08841	0.967	-1972.5353	756.5472
	**E100 10**	814.43552	405.08841	0.761	-550.1057	2178.9768
	**EA 0.01**	192.86158	405.08841	1.000	-1171.6797	1557.4028
	**EA 1.0**	345.22901	405.08841	1.000	-1019.3123	1709.7703
	**EA 10**	-1089.43547	405.08841	0.288	-2453.9767	275.1058
**EA 1.0**	**E25 0.01**	264.75685	452.90261	1.000	-1260.8467	1790.3604
	**E25 0.1**	-1435.48474	452.90261	0.090	-2961.0883	90.1188
	**E25 1.0**	-7.64554	452.90261	1.000	-1533.2491	1517.9580
	**E25 10**	-1293.56866	452.90261	0.200	-2819.1722	232.0349
	**VPA**	1357.71649*	335.88157	0.005	226.2986	2489.1343
	**DMSO**	320.33072	335.88157	1.000	-811.0871	1451.7486
	**E100 0.01**	190.76199	405.08841	1.000	-1173.7793	1555.3033
	**E100 0.1**	436.14499	405.08841	0.999	-928.3963	1800.6863
	**E100 1.0**	-953.22305	405.08841	0.516	-2317.7643	411.3182
	**E100 10**	469.20652	405.08841	0.997	-895.3347	1833.7478

	**EA 0.01**	-152.36743	405.08841	1.000	-1516.9087	1212.1738
	**EA 0.1**	-345.22901	405.08841	1.000	-1709.7703	1019.3123
	**EA 10**	-1434.66448*	405.08841	0.029	-2799.2057	-70.1232
**EA 10**	**E25 0.01**	1699.42132*	452.90261	0.014	173.8178	3225.0248
	**E25 0.1**	-0.82026	452.90261	1.000	-1526.4238	1524.7833
	**E25 1.0**	1427.01894	452.90261	0.095	-98.5846	2952.6225
	**E25 10**	141.09582	452.90261	1.000	-1384.5077	1666.6993
	**VPA**	2792.38097*	335.88157	0.000	1660.9631	3923.7988
	**DMSO**	1754.99520*	335.88157	0.000	623.5774	2886.4130
	**E100 0.01**	1625.42647*	405.08841	0.005	260.8852	2989.9677
	**E100 0.1**	1870.80947*	405.08841	0.000	506.2682	3235.3507
	**E100 1.0**	481.44143	405.08841	0.996	-883.0998	1845.9827
	**E100 10**	1903.87099*	405.08841	0.000	539.3297	3268.4123
	**EA 0.01**	1282.29705	405.08841	0.091	-82.2442	2646.8383
	**EA 0.1**	1089.43547	405.08841	0.288	-275.1058	2453.9767
	**EA 1.0**	1434.66448*	405.08841	0.029	70.1232	2799.2057
**CAN-3**						
**E25 0.01**	**E25 0.1**	62.16452	424.57264	1.000	-1367.2908	1491.6199
	**E25 1.0**	-56.95653	424.57264	1.000	-1486.4119	1372.4988
	**E25 10**	-2167.53087*	424.57264	0.000	-3596.9862	-738.0755
	**VPA**	1139.33891	346.66211	0.064	-27.8065	2306.4843
	**DMSO**	-175.87069	346.66211	1.000	-1343.0161	991.2747
	**E100 0.01**	-16.55614	424.57264	1.000	-1446.0115	1412.8992
	**E100 0.1**	-494.97952	424.57264	0.997	-1924.4349	934.4759
	**E100 1.0**	-1993.48263*	424.57264	0.000	-3422.9380	-564.0273
	**E100 10**	457.24210	424.57264	0.999	-972.2133	1886.6975
	**EA 0.01**	612.08461	424.57264	0.977	-817.3708	2041.5400
	**EA 0.1**	556.11269	424.57264	0.990	-873.3427	1985.5681
	**EA 1.0**	1200.85478	424.57264	0.213	-228.6006	2630.3102
	**EA 10**	-1410.93887	424.57264	0.057	-2840.3942	18.5165
**E25 0.1**	**E25 0.01**	-62.16452	424.57264	1.000	-1491.6199	1367.2908
	**E25 1.0**	-119.12106	424.57264	1.000	-1548.5764	1310.3343
	**E25 10**	-2229.69540*	424.57264	0.000	-3659.1508	-800.2400
	**VPA**	1077.17439	346.66211	0.106	-89.9710	2244.3198
	**DMSO**	-238.03521	346.66211	1.000	-1405.1806	929.1102
	**E100 0.01**	-78.72066	424.57264	1.000	-1508.1760	1350.7347
	**E100 0.1**	-557.14404	424.57264	0.990	-1986.5994	872.3113
	**E100 1.0**	-2055.64715*	424.57264	0.000	-3485.1025	-626.1918
	**E100 10**	395.07758	424.57264	1.000	-1034.3778	1824.5330
	**EA 0.01**	549.92009	424.57264	0.991	-879.5353	1979.3755
	**EA 0.1**	493.94817	424.57264	0.997	-935.5072	1923.4035
	**EA 1.0**	1138.69025	424.57264	0.292	-290.7651	2568.1456
	**EA 10**	-1473.10340*	424.57264	0.036	-2902.5588	-43.6480
**E25 1.0**	**E25 0.01**	56.95653	424.57264	1.000	-1372.4988	1486.4119
	**E25 0.1**	119.12106	424.57264	1.000	-1310.3343	1548.5764
	**E25 10**	-2110.57434*	424.57264	0.000	-3540.0297	-681.1190
	**VPA**	1196.29545*	346.66211	0.038	29.1500	2363.4409
	**DMSO**	-118.91415	346.66211	1.000	-1286.0596	1048.2313
	**E100 0.01**	40.40040	424.57264	1.000	-1389.0550	1469.8558
	**E100 0.1**	-438.02299	424.57264	0.999	-1867.4784	991.4324
	**E100 1.0**	-1936.52609*	424.57264	0.001	-3365.9815	-507.0707
	**E100 10**	514.19864	424.57264	0.995	-915.2567	1943.6540
	**EA 0.01**	669.04115	424.57264	0.952	-760.4142	2098.4965
	**EA 0.1**	613.06923	424.57264	0.976	-816.3861	2042.5246
	**EA 1.0**	1257.81131	424.57264	0.155	-171.6441	2687.2667
	**EA 10**	-1353.98234	424.57264	0.085	-2783.4377	75.4730
**E25 10**	**E25 0.01**	2167.53087*	424.57264	0.000	738.0755	3596.9862
	**E25 0.1**	2229.69540*	424.57264	0.000	800.2400	3659.1508
	**E25 1.0**	2110.57434*	424.57264	0.000	681.1190	3540.0297
	**VPA**	3306.86979*	346.66211	0.000	2139.7244	4474.0152
	**DMSO**	1991.66018*	346.66211	0.000	824.5148	3158.8056
	**E100 0.01**	2150.97473*	424.57264	0.000	721.5194	3580.4301

	**E100 0.1**	1672.55135*	424.57264	0.007	243.0960	3102.0067
	**E100 1.0**	174.04824	424.57264	1.000	-1255.4071	1603.5036
	**E100 10**	2624.77297*	424.57264	0.000	1195.3176	4054.2283
	**EA 0.01**	2779.61548*	424.57264	0.000	1350.1601	4209.0709
	**EA 0.1**	2723.64356*	424.57264	0.000	1294.1882	4153.0989
	**EA 1.0**	3368.38565*	424.57264	0.000	1938.9303	4797.8410
	**EA 10**	756.59200	424.57264	0.884	-672.8634	2186.0474
**VPA**	**E25 0.01**	-1139.33891	346.66211	0.064	-2306.4843	27.8065
	**E25 0.1**	-1077.17439	346.66211	0.106	-2244.3198	89.9710
	**E25 1.0**	-1196.29545*	346.66211	0.038	-2363.4409	-29.1500
	**E25 10**	-3306.86979*	346.66211	0.000	-4474.0152	-2139.7244
	**DMSO**	-1315.20960*	245.12713	0.000	-2140.5060	-489.9132
	**E100 0.01**	-1155.89505	346.66211	0.055	-2323.0405	11.2504
	**E100 0.1**	-1634.31843*	346.66211	0.000	-2801.4639	-467.1730
	**E100 1.0**	-3132.82154*	346.66211	0.000	-4299.9670	-1965.6761
	**E100 10**	-682.09681	346.66211	0.787	-1849.2422	485.0486
	**EA 0.01**	-527.25430	346.66211	0.964	-1694.3997	639.8911
	**EA 0.1**	-583.22622	346.66211	0.922	-1750.3716	583.9192
	**EA 1.0**	61.51586	346.66211	1.000	-1105.6296	1228.6613
	**EA 10**	-2550.27779*	346.66211	0.000	-3717.4232	-1383.1324
**DMSO**	**E25 0.01**	175.87069	346.66211	1.000	-991.2747	1343.0161
	**E25 0.1**	238.03521	346.66211	1.000	-929.1102	1405.1806
	**E25 1.0**	118.91415	346.66211	1.000	-1048.2313	1286.0596
	**E25 10**	-1991.66018*	346.66211	0.000	-3158.8056	-824.5148
	**VPA**	1315.20960*	245.12713	0.000	489.9132	2140.5060
	**E100 0.01**	159.31455	346.66211	1.000	-1007.8309	1326.4600
	**E100 0.1**	-319.10883	346.66211	1.000	-1486.2543	848.0366
	**E100 1.0**	-1817.61194*	346.66211	0.000	-2984.7574	-650.4665
	**E100 10**	633.11279	346.66211	0.864	-534.0326	1800.2582
	**EA 0.01**	787.95530	346.66211	0.576	-379.1901	1955.1007
	**EA 0.1**	731.98338	346.66211	0.693	-435.1620	1899.1288
	**EA 1.0**	1376.72546*	346.66211	0.006	209.5800	2543.8709
	**EA 10**	-1235.06819*	346.66211	0.027	-2402.2136	-67.9228
**E100 0.01**	**E25 0.01**	16.55614	424.57264	1.000	-1412.8992	1446.0115
	**E25 0.1**	78.72066	424.57264	1.000	-1350.7347	1508.1760
	**E25 1.0**	-40.40040	424.57264	1.000	-1469.8558	1389.0550
	**E25 10**	-2150.97473*	424.57264	0.000	-3580.4301	-721.5194
	**VPA**	1155.89505	346.66211	0.055	-11.2504	2323.0405
	**DMSO**	-159.31455	346.66211	1.000	-1326.4600	1007.8309
	**E100 0.1**	-478.42338	424.57264	0.998	-1907.8788	951.0320
	**E100 1.0**	-1976.92649*	424.57264	0.000	-3406.3819	-547.4711
	**E100 10**	473.79824	424.57264	0.998	-955.6571	1903.2536
	**EA 0.01**	628.64075	424.57264	0.971	-800.8146	2058.0961
	**EA 0.1**	572.66883	424.57264	0.987	-856.7865	2002.1242
	**EA 1.0**	1217.41092	424.57264	0.195	-212.0445	2646.8663
	**EA 10**	-1394.38273	424.57264	0.064	-2823.8381	35.0726
**E100 0.1**	**E25 0.01**	494.97952	424.57264	0.997	-934.4759	1924.4349
	**E25 0.1**	557.14404	424.57264	0.990	-872.3113	1986.5994
	**E25 1.0**	438.02299	424.57264	0.999	-991.4324	1867.4784
	**E25 10**	-1672.55135*	424.57264	0.007	-3102.0067	-243.0960
	**VPA**	1634.31843*	346.66211	0.000	467.1730	2801.4639
	**DMSO**	319.10883	346.66211	1.000	-848.0366	1486.2543
	**E100 0.01**	478.42338	424.57264	0.998	-951.0320	1907.8788
	**E100 1.0**	-1498.50311*	424.57264	0.030	-2927.9585	-69.0477
	**E100 10**	952.22162	424.57264	0.598	-477.2337	2381.6770
	**EA 0.01**	1107.06413	424.57264	0.338	-322.3912	2536.5195
	**EA 0.1**	1051.09221	424.57264	0.427	-378.3632	2480.5476
	**EA 1.0**	1695.83430*	424.57264	0.006	266.3789	3125.2897
	**EA 10**	-915.95935	424.57264	0.661	-2345.4147	513.4960
**E100 1.0**	**E25 0.01**	1993.48263*	424.57264	0.000	564.0273	3422.9380
	**E25 0.1**	2055.64715*	424.57264	0.000	626.1918	3485.1025
	**E25 1.0**	1936.52609*	424.57264	0.001	507.0707	3365.9815

	**E25 10**	-174.04824	424.57264	1.000	-1603.5036	1255.4071
	**VPA**	3132.82154*	346.66211	0.000	1965.6761	4299.9670
	**DMSO**	1817.61194*	346.66211	0.000	650.4665	2984.7574
	**E100 0.01**	1976.92649*	424.57264	0.000	547.4711	3406.3819
	**E100 0.1**	1498.50311*	424.57264	0.030	69.0477	2927.9585
	**E100 10**	2450.72473*	424.57264	0.000	1021.2694	3880.1801
	**EA 0.01**	2605.56724*	424.57264	0.000	1176.1119	4035.0226
	**EA 0.1**	2549.59532*	424.57264	0.000	1120.1399	3979.0507
	**EA 1.0**	3194.33741*	424.57264	0.000	1764.8820	4623.7928
	**EA 10**	582.54375	424.57264	0.985	-846.9116	2011.9991
**E100 10**	**E25 0.01**	-457.24210	424.57264	0.999	-1886.6975	972.2133
	**E25 0.1**	-395.07758	424.57264	1.000	-1824.5330	1034.3778
	**E25 1.0**	-514.19864	424.57264	0.995	-1943.6540	915.2567
	**E25 10**	-2624.77297*	424.57264	0.000	-4054.2283	-1195.3176
	**VPA**	682.09681	346.66211	0.787	-485.0486	1849.2422
	**DMSO**	-633.11279	346.66211	0.864	-1800.2582	534.0326
	**E100 0.01**	-473.79824	424.57264	0.998	-1903.2536	955.6571
	**E100 0.1**	-952.22162	424.57264	0.598	-2381.6770	477.2337
	**E100 1.0**	-2450.72473*	424.57264	0.000	-3880.1801	-1021.2694
	**EA 0.01**	154.84251	424.57264	1.000	-1274.6129	1584.2979
	**EA 0.1**	98.87059	424.57264	1.000	-1330.5848	1528.3260
	**EA 1.0**	743.61267	424.57264	0.896	-685.8427	2173.0680
	**EA 10**	-1868.18098*	424.57264	0.001	-3297.6363	-438.7256
**EA 0.01**	**E25 0.01**	-612.08461	424.57264	0.977	-2041.5400	817.3708
	**E25 0.1**	-549.92009	424.57264	0.991	-1979.3755	879.5353
	**E25 1.0**	-669.04115	424.57264	0.952	-2098.4965	760.4142
	**E25 10**	-2779.61548*	424.57264	0.000	-4209.0709	-1350.1601
	**VPA**	527.25430	346.66211	0.964	-639.8911	1694.3997
	**DMSO**	-787.95530	346.66211	0.576	-1955.1007	379.1901
	**E100 0.01**	-628.64075	424.57264	0.971	-2058.0961	800.8146
	**E100 0.1**	-1107.06413	424.57264	0.338	-2536.5195	322.3912
	**E100 1.0**	-2605.56724*	424.57264	0.000	-4035.0226	-1176.1119
	**E100 10**	-154.84251	424.57264	1.000	-1584.2979	1274.6129
	**EA 0.1**	-55.97192	424.57264	1.000	-1485.4273	1373.4835
	**EA 1.0**	588.77016	424.57264	0.983	-840.6852	2018.2255
	**EA 10**	-2023.02349*	424.57264	0.000	-3452.4789	-593.5681
**EA 0.1**	**E25 0.01**	-556.11269	424.57264	0.990	-1985.5681	873.3427
	**E25 0.1**	-493.94817	424.57264	0.997	-1923.4035	935.5072
	**E25 1.0**	-613.06923	424.57264	0.976	-2042.5246	816.3861
	**E25 10**	-2723.64356*	424.57264	0.000	-4153.0989	-1294.1882
	**VPA**	583.22622	346.66211	0.922	-583.9192	1750.3716
	**DMSO**	-731.98338	346.66211	0.693	-1899.1288	435.1620
	**E100 0.01**	-572.66883	424.57264	0.987	-2002.1242	856.7865
	**E100 0.1**	-1051.09221	424.57264	0.427	-2480.5476	378.3632
	**E100 1.0**	-2549.59532*	424.57264	0.000	-3979.0507	-1120.1399
	**E100 10**	-98.87059	424.57264	1.000	-1528.3260	1330.5848
	**EA 0.01**	55.97192	424.57264	1.000	-1373.4835	1485.4273
	**EA 1.0**	644.74208	424.57264	0.964	-784.7133	2074.1975
	**EA 10**	-1967.05157*	424.57264	0.000	-3396.5069	-537.5962
**EA 1.0**	**E25 0.01**	-1200.85478	424.57264	0.213	-2630.3102	228.6006
	**E25 0.1**	-1138.69025	424.57264	0.292	-2568.1456	290.7651
	**E25 1.0**	-1257.81131	424.57264	0.155	-2687.2667	171.6441
	**E25 10**	-3368.38565*	424.57264	0.000	-4797.8410	-1938.9303
	**VPA**	-61.51586	346.66211	1.000	-1228.6613	1105.6296
	**DMSO**	-1376.72546*	346.66211	0.006	-2543.8709	-209.5800
	**E100 0.01**	-1217.41092	424.57264	0.195	-2646.8663	212.0445
	**E100 0.1**	-1695.83430*	424.57264	0.006	-3125.2897	-266.3789
	**E100 1.0**	-3194.33741*	424.57264	0.000	-4623.7928	-1764.8820
	**E100 10**	-743.61267	424.57264	0.896	-2173.0680	685.8427
	**EA 0.01**	-588.77016	424.57264	0.983	-2018.2255	840.6852
	**EA 0.1**	-644.74208	424.57264	0.964	-2074.1975	784.7133
	**EA 10**	-2611.79365*	424.57264	0.000	-4041.2490	-1182.3383
**EA 10**	**E25 0.01**	1410.93887*	424.57264	0.057	-18.5165	2840.3942

**E25 0.1**	1473.10340*	424.57264	0.036	43.6480	2902.5588
**E25 1.0**	1353.98234	424.57264	0.085	-75.4730	2783.4377
**E25 10**	-756.59200	424.57264	0.884	-2186.0474	672.8634
**VPA**	2550.27779*	346.66211	0.000	1383.1324	3717.4232
**DMSO**	1235.06819*	346.66211	0.027	67.9228	2402.2136
**E100 0.01**	1394.38273	424.57264	0.064	-35.0726	2823.8381
**E100 0.1**	915.95935	424.57264	0.661	-513.4960	2345.4147
**E100 1.0**	-582.54375	424.57264	0.985	-2011.9991	846.9116
**E100 10**	1868.18098*	424.57264	0.001	438.7256	3297.6363
**EA 0.01**	2023.02349*	424.57264	0.000	593.5681	3452.4789
**EA 0.1**	1967.05157*	424.57264	0.000	537.5962	3396.5069
**EA 1.0**	2611.79365*	424.57264	0.000	1182.3383	4041.2490

Viability Test (Dark; 3 min)

Table S4. Descriptive Statistics for CAN-1

Test solution	Dose (µg/ml)	N	Condition	Mean	SEM
**E100**	0.01	36	1	128.58	17.36
		36	2	178.99	14.53
**E100**	0.1	36	1	64.79	11.84
		36	2	134.15	14.37
**E100**	1.0	36	1	62.14	14.57
		36	2	136.63	15.89
**E100**	10	36	1	170.22	10.25
		36	2	221.72	14.52
**E25**	0.01	36	1	145.25	19.03
		36	2	198.06	14.72
**E25**	0.1	36	1	88.01	17.34
		36	2	155.43	19.16
**E25**	1.0	36	1	114.98	14.70
		36	2	202.89	17.17
**E25**	10	36	1	133.31	11.26
		36	2	192.27	20.28
**EA**	0.01	36	1	97.90	17.30
		36	2	130.04	16.25
**EA**	0.1	36	1	105.27	17.78
		36	2	133.80	15.50
**EA**	1.0	36	1	79.96	13.46
		36	2	146.73	15.27
**EA**	10	36	1	116.76	16.29
		36	2	174.12	18.56
**DMSO**		108	1	88.17	8.77
		108	2	149.79	8.98
**VPA**		108	1	41.08	6.95
		108	2	98.31	9.54

Note. Condition: 1 = Last min of Light, 2 = First min of Dark

Table S5. T-Tests for CAN-1

					95% Confidence Interval of the Difference				p value	
**Test solution**	**Dose (µg/ml)**	**Differences**	**Mean**	**SEM**	**Lower**	**Upper**	**t**	**df**	**One-Sided**	**Two-Sided**
**E100**	**0.01**	**Light - Dark**	-50.42	14.72	-80.29	-20.54	-3.43	35.00	0.00	0.00
**E100**	**0.1**	**Light - Dark**	-69.36	10.91	-91.51	-47.21	-6.36	35.00	0.00	0.00
**E100**	**1.0**	**Light - Dark**	-74.50	15.41	-105.78	-43.22	-4.83	35.00	0.00	0.00
**E100**	**10**	**Light - Dark**	-51.50	14.76	-81.46	-21.54	-3.49	35.00	0.00	0.00
**E25**	**0.01**	**Light - Dark**	-52.80	15.39	-84.05	-21.56	-3.43	35.00	0.00	0.00
**E25**	**0.1**	**Light - Dark**	-67.43	14.21	-96.27	-38.58	-4.75	35.00	0.00	0.00
**E25**	**1.0**	**Light - Dark**	-87.91	15.16	-118.68	-57.14	-5.80	35.00	0.00	0.00
**E25**	**10**	**Light - Dark**	-58.97	21.11	-101.82	-16.11	-2.79	35.00	0.00	0.01
**EA**	**0.01**	**Light - Dark**	-32.14	16.47	-65.57	1.30	-1.95	35.00	0.03	0.06
**EA**	**0.1**	**Light - Dark**	-28.53	9.42	-47.65	-9.41	-3.03	35.00	0.00	0.00

**EA**	**1.0**	**Light - Dark**	-66.77	17.58	-102.46	-31.08	-3.80	35.00	0.00	0.00
**EA**	**10**	**Light - Dark**	-57.36	14.27	-86.33	-28.40	-4.02	35.00	0.00	0.00
**DMSO**		**Light - Dark**	-61.62	8.02	-77.51	-45.72	-7.68	107.00	0.00	0.00

Table S6. Descriptive Statistics for CAN-2

Test solution	Dose (µg/ml)	N	Condition	Mean	SEM
**E100**	0.01	36	1	142.23	16.14
		36	2	189.52	12.71
**E100**	0.1	36	1	122.70	15.19
		36	2	197.70	12.22
**E100**	1.0	36	1	197.21	17.62
		36	2	222.52	12.39
**E100**	10	36	1	102.54	12.03
		36	2	95.84	11.07
**E25**	0.01	36	1	104.94	19.97
		36	2	225.55	17.89
**E25**	0.1	36	1	206.30	20.87
		36	2	243.13	15.64
**E25**	1.0	36	1	153.78	21.43
		36	2	217.06	16.72
**E25**	10	36	1	137.30	20.33
		36	2	176.32	19.19
**EA**	0.01	36	1	131.89	19.68
		36	2	197.02	14.47
**EA**	0.1	36	1	136.74	16.92
		36	2	192.27	11.58
**EA**	1.0	36	1	142.23	17.80
		36	2	189.42	13.00
**EA**	10	36	1	162.11	12.25
		36	2	189.63	13.18
**DMSO**		108	1	128.74	10.23
		108	2	187.93	8.78
**VPA**		108	1	60.97	7.73
		108	2	131.61	8.98

Note. Condition: 1 = Last min of Light, 2 = First min of Dark

Table S7. T-Tests for CAN-2

				95% Confidence Interval of the Difference				p value	
**Test solution (µg/ml)**	**Differences**	**Mean**	**SEM**	**Lower**	**Upper**	**t**	**df**	**One-Sided**	**Two-Sided**
**E100_0.01**	**Light - Dark**	-47.29	14.37	-76.47	-18.11	-3.29	35.00	0.00	0.00
**E100_0.1**	**Light - Dark**	-75.00	20.65	-116.92	-33.07	-3.63	35.00	0.00	0.00
**E100_1**	**Light - Dark**	-25.32	19.16	-64.20	13.57	-1.32	35.00	0.10	0.19
**E100_10**	**Light - Dark**	6.70	15.48	-24.73	38.13	0.43	35.00	0.33	0.67
**E25_0.01**	**Light - Dark**	-120.61	16.13	-153.97	-87.25	-7.48	23.00	0.00	0.00
**E25_0.1**	**Light - Dark**	-36.83	22.73	-83.85	10.20	-1.62	23.00	0.06	0.12
**E25_1**	**Light - Dark**	-63.28	19.00	-102.58	-23.98	-3.33	23.00	0.00	0.00
**E25_10**	**Light - Dark**	-39.02	15.96	-72.04	-6.01	-2.45	23.00	0.01	0.02

**EA_0.01**	**Light - Dark**	-65.12	16.11	-97.82	-32.42	-4.04	35.00	0.00	0.00
**EA_0.1**	**Light - Dark**	-55.53	15.79	-87.58	-23.47	-3.52	35.00	0.00	0.00
**EA_1**	**Light - Dark**	-47.18	14.25	-76.11	-18.25	-3.31	35.00	0.00	0.00
**EA_10**	**Light - Dark**	-27.52	14.99	-57.94	2.91	-1.84	35.00	0.04	0.07
**DMSO**	**Light - Dark**	-59.19	9.37	-77.79	-40.59	-6.32	95.00	0.00	0.00

Table S8. Descriptive Statistics for CAN-3

Test solution (µg/ml)	N	Condition	Mean	SEM
**E100_0.01**	36	1	131.41	20.22
	36	2	214.03	11.18
**E100_0.1**	36	1	167.67	20.89
	36	2	216.69	17.84
**E100_1**	36	1	185.02	20.53
	36	2	219.91	15.70
**E100_10**	36	1	104.45	15.95
	36	2	152.83	19.03
**E25_0.01**	36	1	133.45	16.55
_	36	2	205.60	10.82
**E250.1**	36	1	134.76	18.65
_	36	2	181.61	15.08
**E251**	36	1	154.56	17.85
_	36	2	199.46	13.17
**E2510**	36	1	138.28	9.09
_	36	2	253.27	14.63
**EA0.01**	36	1	138.77	19.28
_	36	2	166.53	15.02
**EA_0.1**	36	1	120.40	18.76
	36	2	184.19	12.32
**EA_1**	36	1	76.62	15.42
	36	2	169.96	14.42
**EA_10**	36	1	146.51	9.94
	36	2	237.03	10.86
**DMSO**	108	1	142.08	10.58
	108	2	217.43	7.71
**VPA**	108	1	55.69	8.14
	108	2	123.79	8.94

Note. Condition: 1 = Last min of Light, 2 = First min of Dark

Table S9. T-Tests for CAN-3

				**95% Confidence Interval of the Difference**				**p value**	
**Test solution (µg/ml)**	**Differences**	**Mean**	**SEM**	**Lower**	**Upper**	**t**	**df**	**One-Sided**	**Two-Sided**
**E100_0.01**	**Light - Dark**	-82.63	21.41	-126.08	-39.17	-3.86	35.00	0.00	0.00
**E100_0.1**	**Light - Dark**	-49.02	20.34	-90.31	-7.73	-2.41	35.00	0.01	0.02
**E100_1**	**Light - Dark**	-34.89	19.99	-75.46	5.69	-1.75	35.00	0.04	0.09
**E100_10**	**Light - Dark**	-48.38	23.27	-95.63	-1.13	-2.08	35.00	0.02	0.05
**E25_0.01**	**Light - Dark**	-72.15	16.12	-104.88	-39.42	-4.48	35.00	0.00	0.00
**E25_0.1**	**Light - Dark**	-46.85	16.96	-81.27	-12.42	-2.76	35.00	0.00	0.01

**E25_1**	**Light - Dark**	-44.90	15.78	-76.94	-12.85	-2.84	35.00	0.00	0.01
**E25_10**	**Light - Dark**	-114.99	15.60	-146.65	-83.32	-7.37	35.00	0.00	0.00
**EA_0.01**	**Light - Dark**	-27.76	16.66	-61.57	6.06	-1.67	35.00	0.05	0.10
**EA_0.1**	**Light - Dark**	-63.79	15.03	-94.29	-33.29	-4.25	35.00	0.00	0.00
**EA_1**	**Light - Dark**	-93.34	15.88	-125.58	-61.09	-5.88	35.00	0.00	0.00
**EA_10**	**Light - Dark**	-90.52	13.94	-118.83	-62.21	-6.49	35.00	0.00	0.00
**DMSO**	**Light - Dark**	-75.35	10.11	-95.39	-55.31	-7.45	107.00	0.00	0.00

Phase 1

Table S10. Descriptive Statistics

Pre-test solution (30 minutes)	Test solution (30 minutes)	N	Mean	Std. Deviation	Std. Error	95% Confidence Interval for Mean		Minimum	Maximum
						Lower Bound	Upper Bound		
**CBD 6**	**PTZ**	48	7063.60	2678.40	386.59	6285.87	7841.33	2662.07	12465.71
**CBD 9**	**PTZ**	48	4983.18	2155.45	311.11	4357.30	5609.06	1391.82	9925.99
**CBD 12**	**PTZ**	48	2788.68	1694.64	244.60	2296.61	3280.76	550.45	8853.08
**CBD 15**	**PTZ**	48	2201.19	1190.71	171.86	1855.45	2546.94	745.58	5233.26
**PCBD 18**	**PTZ**	48	1373.89	765.41	110.48	1151.64	1596.14	178.00	3510.73
**DMSO**	**DMSO**	48	1782.35	1331.73	192.22	1395.65	2169.04	184.21	5622.06
**DMSO**	**PTZ**	48	7321.36	2457.05	354.64	6607.90	8034.81	3894.24	13928.68
**VPA**	**PTZ**	48	2684.29	1638.60	236.51	2208.49	3160.09	421.48	7242.04

VPA – 5 mM; PTZ - 15 mM

Table S11. Effect Sizes

	Point Estimate	95% Confidence Interval	
		Lower	Upper
**Eta-squared**	0.596	0.530	0.638
**Epsilon-squared**	0.588	0.521	0.631
**Omega-squared Fixed-effect**	0.588	0.521	0.630
**Omega-squared Random-effect**	0.169	0.134	0.196

Table S12. Tukey HSD Post-hoc Tests

Test solution (μg/ml)	Test solution (μg/ml)	Mean Difference	Std. Error	Sig.	95% Confidence Interval	
					Lower Bound	Upper Bound
**CBD 6**	**CBD 9**	2080.42	376.32	0.00	933.36	3227.48
	**CBD 12**	4274.92	376.32	0.00	3127.85	5421.98
	**CBD 15**	4862.41	376.32	0.00	3715.34	6009.47
	**CBD 18**	5689.71	376.32	0.00	4542.65	6836.77
	**DMSO**	5281.25	376.32	0.00	4134.19	6428.32
	**PTZ**	-257.76	376.32	1.00	-1404.82	889.31
	**VPA**	4379.31	376.32	0.00	3232.25	5526.38
**CBD 9**	**CBD 6**	-2080.42	376.32	0.00	-3227.48	-933.36
	**CBD 12**	2194.50	376.32	0.00	1047.43	3341.56
	**CBD 15**	2781.99	376.32	0.00	1634.92	3929.05
	**CBD 18**	3609.29	376.32	0.00	2462.23	4756.35
	**DMSO**	3200.83	376.32	0.00	2053.77	4347.90
	**PTZ**	-2338.18	376.32	0.00	-3485.24	-1191.11
	**VPA**	2298.89	376.32	0.00	1151.83	3445.96
**CBD 12**	**CBD 6**	-4274.92	376.32	0.00	-5421.98	-3127.85
	**CBD 9**	-2194.50	376.32	0.00	-3341.56	-1047.43
	**CBD 15**	587.49	376.32	0.77	-559.57	1734.55
	**CBD 18**	1414.79	376.32	0.00	267.73	2561.86
	**DMSO**	1006.34	376.32	0.13	-140.73	2153.40
	**PTZ**	-4532.67	376.32	0.00	-5679.74	-3385.61

	**VPA**	104.39	376.32	1.00	-1042.67	1251.46
**CBD 15**	**CBD 6**	-4862.41	376.32	0.00	-6009.47	-3715.34
	**CBD 9**	-2781.99	376.32	0.00	-3929.05	-1634.92
	**CBD 12**	-587.49	376.32	0.77	-1734.55	559.57
	**CBD 18**	827.30	376.32	0.36	-319.76	1974.36
	**DMSO**	418.84	376.32	0.95	-728.22	1565.91
	**PTZ**	-5120.16	376.32	0.00	-6267.23	-3973.10
	**VPA**	-483.10	376.32	0.90	-1630.16	663.97
**CBD 18**	**CBD 6**	-5689.71	376.32	0.00	-6836.77	-4542.65
	**CBD 9**	-3609.29	376.32	0.00	-4756.35	-2462.23
	**CBD 12**	-1414.79	376.32	0.00	-2561.86	-267.73
	**CBD 15**	-827.30	376.32	0.36	-1974.36	319.76
	**DMSO**	-408.46	376.32	0.96	-1555.52	738.61
	**PTZ**	-5947.47	376.32	0.00	-7094.53	-4800.40
	**VPA**	-1310.40	376.32	0.01	-2457.46	-163.33
**DMSO**	**CBD 6**	-5281.25	376.32	0.00	-6428.32	-4134.19
	**CBD 9**	-3200.83	376.32	0.00	-4347.90	-2053.77
	**CBD 12**	-1006.34	376.32	0.13	-2153.40	140.73
	**CBD 15**	-418.84	376.32	0.95	-1565.91	728.22
	**CBD 18**	408.46	376.32	0.96	-738.61	1555.52
	**PTZ**	-5539.01	376.32	0.00	-6686.07	-4391.94
	**VPA**	-901.94	376.32	0.25	-2049.00	245.12
**PTZ**	**CBD 6**	257.76	376.32	1.00	-889.31	1404.82
	**CBD 9**	2338.18	376.32	0.00	1191.11	3485.24
	**CBD 12**	4532.67	376.32	0.00	3385.61	5679.74
	**CBD 15**	5120.16	376.32	0.00	3973.10	6267.23
	**CBD 18**	5947.47	376.32	0.00	4800.40	7094.53
	**DMSO**	5539.01	376.32	0.00	4391.94	6686.07
	**VPA**	4637.07	376.32	0.00	3490.00	5784.13
**VPA**	**CBD 6**	-4379.31	376.32	0.00	-5526.38	-3232.25
	**CBD 9**	-2298.89	376.32	0.00	-3445.96	-1151.83
	**CBD 12**	-104.39	376.32	1.00	-1251.46	1042.67
	**CBD 15**	483.10	376.32	0.90	-663.97	1630.16
	**CBD 18**	1310.40	376.32	0.01	163.33	2457.46
	**DMSO**	901.94	376.32	0.25	-245.12	2049.00
	**PTZ**	-4637.07	376.32	0.00	-5784.13	-3490.00

Phase 2

Table S13. Descriptive Statistics

	Test solution (mg/ml)	N	Mean	Std. Deviation	Std. Error	95% Confidence Interval for Mean		Minimum	Maximum
						Lower Bound	Upper Bound		
CAN-1	E25_0.01	36	6871.54	1797.86	299.64	6263.23	7479.85	740.70	9277.28
	E25_0.1	36	7152.18	2173.56	362.26	6416.75	7887.60	690.47	11863.81
	E25_1	36	7221.87	1743.47	290.58	6631.96	7811.77	4202.80	11047.20
	E25_10	36	1568.26	1603.70	267.28	1025.64	2110.87	0.00	6778.17
	E100_0.01	36	7661.54	1598.58	266.43	7120.66	8202.42	4333.24	10720.70
	E100_0.1	36	7453.80	2000.34	333.39	6776.98	8130.62	1772.93	12279.44
	E100_1	36	7997.76	2436.90	406.15	7173.23	8822.29	534.98	14572.22
	E100_10	36	2207.09	1006.12	167.69	1866.67	2547.51	102.67	4276.97
	EA_0.01	36	6719.62	3106.97	517.83	5668.37	7770.87	557.90	11995.49
	EA_0.1	36	7852.10	2381.19	396.86	7046.42	8657.78	1137.37	11841.75
	EA_1	36	6540.11	2727.73	454.62	5617.18	7463.05	552.05	12206.94
	EA_10	36	3064.03	2151.29	358.55	2336.14	3791.92	644.00	8901.62
	DMSO	108	1795.10	1350.97	130.00	1537.39	2052.80	66.25	6898.22
	PTZ	108	7023.64	2699.76	259.78	6508.65	7538.63	613.87	13127.41
	VPA	108	4163.09	2296.32	220.96	3725.05	4601.12	199.12	10940.17
CAN-2	E25_0.01	36	6340.81	1605.59	267.60	5797.56	6884.06	2702.09	9322.57
	E25_0.1	36	6743.81	2114.34	352.39	6028.42	7459.20	3917.13	14288.62
	E25_1	36	6455.11	1626.35	271.06	5904.83	7005.39	3461.84	10550.30
	E25_10	36	2992.77	1815.69	302.62	2378.42	3607.11	562.52	9065.56
	E100_0.01	36	6712.26	2186.40	364.40	5972.49	7452.03	51.43	10316.66
	E100_0.1	36	6911.53	1868.95	311.49	6279.16	7543.89	3051.41	10643.56
	E100_1	36	6323.99	2065.00	344.17	5625.30	7022.69	3137.13	10494.67
	E100_10	36	1825.86	457.04	76.17	1671.22	1980.50	1005.11	3056.22
	EA_0.01	36	6347.97	2318.56	386.43	5563.48	7132.46	1860.01	12060.86
	EA_0.1	36	6755.17	1614.01	269.00	6209.07	7301.28	3162.62	10298.01
	EA_1	36	6627.55	1828.81	304.80	6008.77	7246.33	2873.12	10260.55
	EA_10	36	3460.58	1194.01	199.00	3056.59	3864.58	1459.74	6827.03
	DMSO	108	2903.43	1556.49	149.77	2606.52	3200.34	73.97	7219.78
	PTZ	108	7105.65	1812.85	174.44	6759.84	7451.46	3388.18	12594.49
	VPA	108	4336.59	1649.96	158.77	4021.85	4651.33	1407.16	8368.80
CAN-3	E25_0.01	36	8339.83	2019.04	336.51	7656.69	9022.98	3741.64	12262.05
	E25_0.1	36	7479.08	2489.56	414.93	6636.74	8321.43	0.00	12951.59
	E25_1	36	7603.36	2290.16	381.69	6828.48	8378.23	2502.07	12019.51
	E25_10	36	3475.88	2235.17	372.53	2719.61	4232.15	135.73	7700.95
	E100_0.01	36	8334.64	2550.79	425.13	7471.58	9197.71	2946.99	12656.86
	E100_0.1	36	6839.07	2526.14	421.02	5984.35	7693.79	1044.93	10727.40
	E100_1	36	6510.80	2778.67	463.11	5570.63	7450.97	1077.04	10395.29
	E100_10	36	1266.09	389.24	64.87	1134.39	1397.78	181.41	2107.13
	EA_0.01	36	7915.24	2495.10	415.85	7071.01	8759.46	1950.85	12698.78
	EA_0.1	36	8247.94	1910.37	318.40	7601.56	8894.32	4664.53	11729.84
	EA_1	36	8136.05	1550.54	258.42	7611.42	8660.67	4871.25	11704.71
	EA_10	36	3939.42	2105.32	350.89	3227.08	4651.76	941.26	8907.31

**DMSO**	108	3121.30	1608.00	154.73	2814.56	3428.03	383.18	8173.71
**PTZ**	108	7832.93	2075.45	199.71	7437.03	8228.83	1262.76	12718.48
**VPA**	108	5079.52	2003.38	192.78	4697.37	5461.67	1490.45	10326.11

Table S14. Effect Sizes

		**Point Estimate**	**95% Confidence Interval**	
			**Lower**	**Upper**
**CAN-1**	**Eta-squared**	0.66	0.62	0.68
	**Epsilon-squared**	0.65	0.61	0.67
	**Omega-squared Fixed-effect**	0.65	0.61	0.67
	**Omega-squared Random-effect**	0.10	0.08	0.11
**CAN-2**	**Eta-squared**	0.68	0.64	0.70
	**Epsilon-squared**	0.67	0.63	0.69
	**Omega-squared Fixed-effect**	0.67	0.63	0.69
	**Omega-squared Random-effect**	0.11	0.09	0.12
**CAN-3**	**Eta-squared**	0.68	0.64	0.70
	**Epsilon-squared**	0.67	0.63	0.69
	**Omega-squared Fixed-effect**	0.67	0.63	0.69
	**Omega-squared Random-effect**	0.11	0.09	0.12

Table S15. Tukey HSD Post-hoc Tests

	**Test solution (µg/ml)**	**Mean Difference**	**Std. Error**	**Sig.**	**95% Confidence Interval**	
**CAN-1 (µg/ml)**					**Lower Bound**	**Upper Bound**
**E25_0.01**	**E25_0.01**	-280.64	475.96	1.00	-1946.31	1385.04
	**E25_0.1**	-350.33	475.96	1.00	-2016.01	1315.35
	**E25_1**	5303.28	475.96	0.00	3637.60	6968.96
	**E25_10**	-790.00	475.96	0.97	-2455.68	875.67
	**E100_0.01**	-582.26	475.96	1.00	-2247.94	1083.41
	**E100_0.1**	-1126.22	475.96	0.63	-2791.90	539.46
	**E100_1**	4664.44	475.96	0.00	2998.77	6330.12
	**E100_10**	151.92	475.96	1.00	-1513.76	1817.59
	**EA_0.01**	-980.57	475.96	0.84	-2646.24	685.11
	**EA_0.1**	331.42	475.96	1.00	-1334.25	1997.10
	**EA_1**	3807.51	475.96	0.00	2141.83	5473.19
	**EA_10**	5076.44	388.62	0.00	3716.42	6436.46
	**PTZ**	-152.10	388.62	1.00	-1512.12	1207.91
	**VPA**	2708.45	388.62	0.00	1348.43	4068.47
**E25_0.1**	**E25_0.01**	280.64	475.96	1.00	-1385.04	1946.31
	**E25_0.1**	-69.69	475.96	1.00	-1735.37	1595.99
	**E25_1**	5583.92	475.96	0.00	3918.24	7249.59
	**E25_10**	-509.37	475.96	1.00	-2175.04	1156.31
	**E100_0.01**	-301.62	475.96	1.00	-1967.30	1364.05
	**E100_0.1**	-845.58	475.96	0.95	-2511.26	820.09
	**E100_1**	4945.08	475.96	0.00	3279.41	6610.76
	**E100_10**	432.55	475.96	1.00	-1233.12	2098.23
	**EA_0.01**	-699.93	475.96	0.99	-2365.60	965.75
	**EA_0.1**	612.06	475.96	1.00	-1053.62	2277.74

	**EA_1**	4088.15	475.96	0.00	2422.47	5753.82
	**EA_10**	5357.08	388.62	0.00	3997.06	6717.10
	**PTZ**	128.53	388.62	1.00	-1231.49	1488.55
	**VPA**	2989.09	388.62	0.00	1629.07	4349.11
**E25_1**	**E25_0.01**	350.33	475.96	1.00	-1315.35	2016.01
	**E25_0.1**	69.69	475.96	1.00	-1595.99	1735.37
	**E25_1**	5653.61	475.96	0.00	3987.93	7319.28
	**E25_10**	-439.68	475.96	1.00	-2105.35	1226.00
	**E100_0.01**	-231.93	475.96	1.00	-1897.61	1433.74
	**E100_0.1**	-775.89	475.96	0.98	-2441.57	889.79
	**E100_1**	5014.77	475.96	0.00	3349.10	6680.45
	**E100_10**	502.24	475.96	1.00	-1163.43	2167.92
	**EA_0.01**	-630.24	475.96	1.00	-2295.91	1035.44
	**EA_0.1**	681.75	475.96	0.99	-983.92	2347.43
	**EA_1**	4157.84	475.96	0.00	2492.16	5823.51
	**EA_10**	5426.77	388.62	0.00	4066.75	6786.79
	**PTZ**	198.22	388.62	1.00	-1161.80	1558.24
	**VPA**	3058.78	388.62	0.00	1698.76	4418.80
**E25_10**	**E25_0.01**	-5303.28	475.96	0.00	-6968.96	-3637.60
	**E25_0.1**	-5583.92	475.96	0.00	-7249.59	-3918.24
	**E25_1**	-5653.61	475.96	0.00	-7319.28	-3987.93
	**E25_10**	-6093.28	475.96	0.00	-7758.96	-4427.61
	**E100_0.01**	-5885.54	475.96	0.00	-7551.22	-4219.86
	**E100_0.1**	-6429.50	475.96	0.00	-8095.18	-4763.82
	**E100_1**	-638.83	475.96	1.00	-2304.51	1026.84
	**E100_10**	-5151.36	475.96	0.00	-6817.04	-3485.69
	**EA_0.01**	-6283.84	475.96	0.00	-7949.52	-4618.17
	**EA_0.1**	-4971.86	475.96	0.00	-6637.53	-3306.18
	**EA_1**	-1495.77	475.96	0.14	-3161.45	169.91
	**EA_10**	-226.84	388.62	1.00	-1586.86	1133.18
	**PTZ**	-5455.38	388.62	0.00	-6815.40	-4095.36
	**VPA**	-2594.83	388.62	0.00	-3954.85	-1234.81
**E100_0.01**	**E25_0.01**	790.00	475.96	0.97	-875.67	2455.68
	**E25_0.1**	509.37	475.96	1.00	-1156.31	2175.04
	**E25_1**	439.68	475.96	1.00	-1226.00	2105.35
	**E25_10**	6093.28	475.96	0.00	4427.61	7758.96
	**E100_0.01**	207.74	475.96	1.00	-1457.93	1873.42
	**E100_0.1**	-336.22	475.96	1.00	-2001.89	1329.46
	**E100_1**	5454.45	475.96	0.00	3788.77	7120.13
	**E100_10**	941.92	475.96	0.88	-723.76	2607.60
	**EA_0.01**	-190.56	475.96	1.00	-1856.24	1475.12
	**EA_0.1**	1121.43	475.96	0.64	-544.25	2787.10
	**EA_1**	4597.51	475.96	0.00	2931.84	6263.19
	**EA_10**	5866.44	388.62	0.00	4506.42	7226.46
	**PTZ**	637.90	388.62	0.98	-722.12	1997.92
	**VPA**	3498.46	388.62	0.00	2138.44	4858.48
**E100_0.1**	**E25_0.01**	582.26	475.96	1.00	-1083.41	2247.94
	**E25_0.1**	301.62	475.96	1.00	-1364.05	1967.30

	**E25_1**	231.93	475.96	1.00	-1433.74	1897.61
	**E25_10**	5885.54	475.96	0.00	4219.86	7551.22
	**E100_0.01**	-207.74	475.96	1.00	-1873.42	1457.93
	**E100_0.1**	-543.96	475.96	1.00	-2209.64	1121.72
	**E100_1**	5246.71	475.96	0.00	3581.03	6912.38
	**E100_10**	734.18	475.96	0.99	-931.50	2399.85
	**EA_0.01**	-398.30	475.96	1.00	-2063.98	1267.37
	**EA_0.1**	913.69	475.96	0.90	-751.99	2579.36
	**EA_1**	4389.77	475.96	0.00	2724.09	6055.45
	**EA_10**	5658.70	388.62	0.00	4298.68	7018.72
	**PTZ**	430.16	388.62	1.00	-929.86	1790.18
	**VPA**	3290.71	388.62	0.00	1930.69	4650.73
**E100_1**	**E25_0.01**	1126.22	475.96	0.63	-539.46	2791.90
	**E25_0.1**	845.58	475.96	0.95	-820.09	2511.26
	**E25_1**	775.89	475.96	0.98	-889.79	2441.57
	**E25_10**	6429.50	475.96	0.00	4763.82	8095.18
	**E100_0.01**	336.22	475.96	1.00	-1329.46	2001.89
	**E100_0.1**	543.96	475.96	1.00	-1121.72	2209.64
	**E100_1**	5790.67	475.96	0.00	4124.99	7456.34
	**E100_10**	1278.14	475.96	0.39	-387.54	2943.81
	**EA_0.01**	145.66	475.96	1.00	-1520.02	1811.33
	**EA_0.1**	1457.64	475.96	0.17	-208.03	3123.32
	**EA_1**	4933.73	475.96	0.00	3268.05	6599.41
	**EA_10**	6202.66	388.62	0.00	4842.64	7562.68
	**PTZ**	974.12	388.62	0.53	-385.90	2334.13
	**VPA**	3834.67	388.62	0.00	2474.65	5194.69
**E100_10**	**E25_0.01**	-4664.44	475.96	0.00	-6330.12	-2998.77
	**E25_0.1**	-4945.08	475.96	0.00	-6610.76	-3279.41
	**E25_1**	-5014.77	475.96	0.00	-6680.45	-3349.10
	**E25_10**	638.83	475.96	1.00	-1026.84	2304.51
	**E100_0.01**	-5454.45	475.96	0.00	-7120.13	-3788.77
	**E100_0.1**	-5246.71	475.96	0.00	-6912.38	-3581.03
	**E100_1**	-5790.67	475.96	0.00	-7456.34	-4124.99
	**E100_10**	-4512.53	475.96	0.00	-6178.21	-2846.85
	**EA_0.01**	-5645.01	475.96	0.00	-7310.69	-3979.33
	**EA_0.1**	-4333.02	475.96	0.00	-5998.70	-2667.34
	**EA_1**	-856.94	475.96	0.94	-2522.61	808.74
	**EA_10**	411.99	388.62	1.00	-948.03	1772.01
	**PTZ**	-4816.55	388.62	0.00	-6176.57	-3456.53
	**VPA**	-1955.99	388.62	0.00	-3316.01	-595.97
**EA_0.01**	**E25_0.01**	-151.92	475.96	1.00	-1817.59	1513.76
	**E25_0.1**	-432.55	475.96	1.00	-2098.23	1233.12
	**E25_1**	-502.24	475.96	1.00	-2167.92	1163.43
	**E25_10**	5151.36	475.96	0.00	3485.69	6817.04
	**E100_0.01**	-941.92	475.96	0.88	-2607.60	723.76
	**E100_0.1**	-734.18	475.96	0.99	-2399.85	931.50
	**E100_1**	-1278.14	475.96	0.39	-2943.81	387.54
	**E100_10**	4512.53	475.96	0.00	2846.85	6178.21

	**EA_0.01**	-1132.48	475.96	0.62	-2798.16	533.20
	**EA_0.1**	179.51	475.96	1.00	-1486.17	1845.18
	**EA_1**	3655.59	475.96	0.00	1989.92	5321.27
	**EA_10**	4924.52	388.62	0.00	3564.50	6284.54
	**PTZ**	-304.02	388.62	1.00	-1664.04	1056.00
	**VPA**	2556.54	388.62	0.00	1196.52	3916.56
**EA_0.1**	**E25_0.01**	980.57	475.96	0.84	-685.11	2646.24
	**E25_0.1**	699.93	475.96	0.99	-965.75	2365.60
	**E25_1**	630.24	475.96	1.00	-1035.44	2295.91
	**E25_10**	6283.84	475.96	0.00	4618.17	7949.52
	**E100_0.01**	190.56	475.96	1.00	-1475.12	1856.24
	**E100_0.1**	398.30	475.96	1.00	-1267.37	2063.98
	**E100_1**	-145.66	475.96	1.00	-1811.33	1520.02
	**E100_10**	5645.01	475.96	0.00	3979.33	7310.69
	**EA_0.01**	1132.48	475.96	0.62	-533.20	2798.16
	**EA_0.1**	1311.99	475.96	0.34	-353.69	2977.67
	**EA_1**	4788.07	475.96	0.00	3122.40	6453.75
	**EA_10**	6057.00	388.62	0.00	4696.98	7417.02
	**PTZ**	828.46	388.62	0.80	-531.56	2188.48
	**VPA**	3689.02	388.62	0.00	2329.00	5049.04
**EA_1**	**E25_0.01**	-331.42	475.96	1.00	-1997.10	1334.25
	**E25_0.1**	-612.06	475.96	1.00	-2277.74	1053.62
	**E25_1**	-681.75	475.96	0.99	-2347.43	983.92
	**E25_10**	4971.86	475.96	0.00	3306.18	6637.53
	**E100_0.01**	-1121.43	475.96	0.64	-2787.10	544.25
	**E100_0.1**	-913.69	475.96	0.90	-2579.36	751.99
	**E100_1**	-1457.64	475.96	0.17	-3123.32	208.03
	**E100_10**	4333.02	475.96	0.00	2667.34	5998.70
	**EA_0.01**	-179.51	475.96	1.00	-1845.18	1486.17
	**EA_0.1**	-1311.99	475.96	0.34	-2977.67	353.69
	**EA_1**	3476.09	475.96	0.00	1810.41	5141.76
	**EA_10**	4745.02	388.62	0.00	3385.00	6105.03
	**PTZ**	-483.53	388.62	1.00	-1843.55	876.49
	**VPA**	2377.03	388.62	0.00	1017.01	3737.05
**EA_10**	**E25_0.01**	-3807.51	475.96	0.00	-5473.19	-2141.83
	**E25_0.1**	-4088.15	475.96	0.00	-5753.82	-2422.47
	**E25_1**	-4157.84	475.96	0.00	-5823.51	-2492.16
	**E25_10**	1495.77	475.96	0.14	-169.91	3161.45
	**E100_0.01**	-4597.51	475.96	0.00	-6263.19	-2931.84
	**E100_0.1**	-4389.77	475.96	0.00	-6055.45	-2724.09
	**E100_1**	-4933.73	475.96	0.00	-6599.41	-3268.05
	**E100_10**	856.94	475.96	0.94	-808.74	2522.61
	**EA_0.01**	-3655.59	475.96	0.00	-5321.27	-1989.92
	**EA_0.1**	-4788.07	475.96	0.00	-6453.75	-3122.40
	**EA_1**	-3476.09	475.96	0.00	-5141.76	-1810.41
	**EA_10**	1268.93	388.62	0.10	-91.09	2628.95
	**PTZ**	-3959.61	388.62	0.00	-5319.63	-2599.59
	**VPA**	-1099.06	388.62	0.30	-2459.08	260.96

**DMSO**	**E25_0.01**	-5076.44	388.62	0.00	-6436.46	-3716.42
	**E25_0.1**	-5357.08	388.62	0.00	-6717.10	-3997.06
	**E25_1**	-5426.77	388.62	0.00	-6786.79	-4066.75
	**E25_10**	226.84	388.62	1.00	-1133.18	1586.86
	**E100_0.01**	-5866.44	388.62	0.00	-7226.46	-4506.42
	**E100_0.1**	-5658.70	388.62	0.00	-7018.72	-4298.68
	**E100_1**	-6202.66	388.62	0.00	-7562.68	-4842.64
	**E100_10**	-411.99	388.62	1.00	-1772.01	948.03
	**EA_0.01**	-4924.52	388.62	0.00	-6284.54	-3564.50
	**EA_0.1**	-6057.00	388.62	0.00	-7417.02	-4696.98
	**EA_1**	-4745.02	388.62	0.00	-6105.03	-3385.00
	**EA_10**	-1268.93	388.62	0.10	-2628.95	91.09
	**PTZ**	-5228.54	274.79	0.00	-6190.22	-4266.86
	**VPA**	-2367.99	274.79	0.00	-3329.67	-1406.31
**PTZ**	**E25_0.01**	152.10	388.62	1.00	-1207.91	1512.12
	**E25_0.1**	-128.53	388.62	1.00	-1488.55	1231.49
	**E25_1**	-198.22	388.62	1.00	-1558.24	1161.80
	**E25_10**	5455.38	388.62	0.00	4095.36	6815.40
	**E100_0.01**	-637.90	388.62	0.98	-1997.92	722.12
	**E100_0.1**	-430.16	388.62	1.00	-1790.18	929.86
	**E100_1**	-974.12	388.62	0.53	-2334.13	385.90
	**E100_10**	4816.55	388.62	0.00	3456.53	6176.57
	**EA_0.01**	304.02	388.62	1.00	-1056.00	1664.04
	**EA_0.1**	-828.46	388.62	0.80	-2188.48	531.56
	**EA_1**	483.53	388.62	1.00	-876.49	1843.55
	**EA_10**	3959.61	388.62	0.00	2599.59	5319.63
	**DMSO**	5228.54	274.79	0.00	4266.86	6190.22
	**VPA**	2860.56	274.79	0.00	1898.88	3822.24
**VPA**	**E25_0.01**	-2708.45	388.62	0.00	-4068.47	-1348.43
	**E25_0.1**	-2989.09	388.62	0.00	-4349.11	-1629.07
	**E25_1**	-3058.78	388.62	0.00	-4418.80	-1698.76
	**E25_10**	2594.83	388.62	0.00	1234.81	3954.85
	**E100_0.01**	-3498.46	388.62	0.00	-4858.48	-2138.44
	**E100_0.1**	-3290.71	388.62	0.00	-4650.73	-1930.69
	**E100_1**	-3834.67	388.62	0.00	-5194.69	-2474.65
	**E100_10**	1955.99	388.62	0.00	595.97	3316.01
	**EA_0.01**	-2556.54	388.62	0.00	-3916.56	-1196.52
	**EA_0.1**	-3689.02	388.62	0.00	-5049.04	-2329.00
	**EA_1**	-2377.03	388.62	0.00	-3737.05	-1017.01
	**EA_10**	1099.06	388.62	0.30	-260.96	2459.08
	**DMSO**	2367.99	274.79	0.00	1406.31	3329.67
	**PTZ**	-2860.56	274.79	0.00	-3822.24	-1898.88
**CAN-2**						
**E25_0.01**	**E25_0.01**	-403.00	384.78	1.00	-1749.60	943.59
	**E25_0.1**	-114.30	384.78	1.00	-1460.90	1232.29
	**E25_1**	3348.04	384.78	0.00	2001.45	4694.64
	**E25_10**	-371.45	384.78	1.00	-1718.05	975.14
	**E100_0.01**	-570.72	384.78	0.99	-1917.31	775.88

	**E100_0.1**	16.82	384.78	1.00	-1329.78	1363.41
	**E100_1**	4514.95	384.78	0.00	3168.35	5861.54
	**E100_10**	-7.16	384.78	1.00	-1353.75	1339.44
	**EA_0.01**	-414.36	384.78	1.00	-1760.96	932.23
	**EA_0.1**	-286.74	384.78	1.00	-1633.34	1059.85
	**EA_1**	2880.23	384.78	0.00	1533.63	4226.82
	**EA_10**	3437.38	314.17	0.00	2337.89	4536.87
	**PTZ**	-764.84	314.17	0.58	-1864.33	334.65
	**VPA**	2004.22	314.17	0.00	904.73	3103.71
**E25_0.1**	**E25_0.01**	403.00	384.78	1.00	-943.59	1749.60
	**E25_0.1**	288.70	384.78	1.00	-1057.89	1635.30
	**E25_1**	3751.04	384.78	0.00	2404.45	5097.64
	**E25_10**	31.55	384.78	1.00	-1315.05	1378.14
	**E100_0.01**	-167.72	384.78	1.00	-1514.31	1178.88
	**E100_0.1**	419.82	384.78	1.00	-926.78	1766.41
	**E100_1**	4917.95	384.78	0.00	3571.35	6264.54
	**E100_10**	395.84	384.78	1.00	-950.75	1742.44
	**EA_0.01**	-11.36	384.78	1.00	-1357.96	1335.23
	**EA_0.1**	116.26	384.78	1.00	-1230.34	1462.85
	**EA_1**	3283.23	384.78	0.00	1936.63	4629.82
	**EA_10**	3840.38	314.17	0.00	2740.89	4939.87
	**PTZ**	-361.84	314.17	1.00	-1461.33	737.65
	**VPA**	2407.22	314.17	0.00	1307.73	3506.71
**E25_1**	**E25_0.01**	114.30	384.78	1.00	-1232.29	1460.90
	**E25_0.1**	-288.70	384.78	1.00	-1635.30	1057.89
	**E25_1**	3462.34	384.78	0.00	2115.75	4808.94
	**E25_10**	-257.15	384.78	1.00	-1603.75	1089.44
	**E100_0.01**	-456.42	384.78	1.00	-1803.01	890.18
	**E100_0.1**	131.12	384.78	1.00	-1215.48	1477.71
	**E100_1**	4629.25	384.78	0.00	3282.65	5975.84
	**E100_10**	107.14	384.78	1.00	-1239.45	1453.74
	**EA_0.01**	-300.06	384.78	1.00	-1646.66	1046.53
	**EA_0.1**	-172.44	384.78	1.00	-1519.04	1174.15
	**EA_1**	2994.53	384.78	0.00	1647.93	4341.12
	**EA_10**	3551.68	314.17	0.00	2452.19	4651.17
	**PTZ**	-650.54	314.17	0.83	-1750.03	448.95
	**VPA**	2118.52	314.17	0.00	1019.03	3218.01
**E25_10**	**E25_0.01**	-3348.04	384.78	0.00	-4694.64	-2001.45
	**E25_0.1**	-3751.04	384.78	0.00	-5097.64	-2404.45
	**E25_1**	-3462.34	384.78	0.00	-4808.94	-2115.75
	**E25_10**	-3719.49	384.78	0.00	-5066.09	-2372.90
	**E100_0.01**	-3918.76	384.78	0.00	-5265.35	-2572.16
	**E100_0.1**	-3331.22	384.78	0.00	-4677.82	-1984.63
	**E100_1**	1166.91	384.78	0.19	-179.69	2513.50
	**E100_10**	-3355.20	384.78	0.00	-4701.80	-2008.61
	**EA_0.01**	-3762.41	384.78	0.00	-5109.00	-2415.81
	**EA_0.1**	-3634.79	384.78	0.00	-4981.38	-2288.19
	**EA_1**	-467.82	384.78	1.00	-1814.41	878.78

	**EA_10**	89.33	314.17	1.00	-1010.16	1188.82
	**PTZ**	-4112.89	314.17	0.00	-5212.38	-3013.40
	**VPA**	-1343.82	314.17	0.00	-2443.31	-244.33
**E100_0.01**	**E25_0.01**	371.45	384.78	1.00	-975.14	1718.05
	**E25_0.1**	-31.55	384.78	1.00	-1378.14	1315.05
	**E25_1**	257.15	384.78	1.00	-1089.44	1603.75
	**E25_10**	3719.49	384.78	0.00	2372.90	5066.09
	**E100_0.01**	-199.27	384.78	1.00	-1545.86	1147.33
	**E100_0.1**	388.27	384.78	1.00	-958.33	1734.87
	**E100_1**	4886.40	384.78	0.00	3539.80	6232.99
	**E100_10**	364.29	384.78	1.00	-982.30	1710.89
	**EA_0.01**	-42.91	384.78	1.00	-1389.51	1303.68
	**EA_0.1**	84.71	384.78	1.00	-1261.89	1431.30
	**EA_1**	3251.68	384.78	0.00	1905.08	4598.27
	**EA_10**	3808.83	314.17	0.00	2709.34	4908.32
	**PTZ**	-393.39	314.17	1.00	-1492.88	706.10
	**VPA**	2375.67	314.17	0.00	1276.18	3475.16
**E100_0.1**	**E25_0.01**	570.72	384.78	0.99	-775.88	1917.31
	**E25_0.1**	167.72	384.78	1.00	-1178.88	1514.31
	**E25_1**	456.42	384.78	1.00	-890.18	1803.01
	**E25_10**	3918.76	384.78	0.00	2572.16	5265.35
	**E100_0.01**	199.27	384.78	1.00	-1147.33	1545.86
	**E100_0.1**	587.54	384.78	0.99	-759.06	1934.13
	**E100_1**	5085.66	384.78	0.00	3739.07	6432.26
	**E100_10**	563.56	384.78	0.99	-783.04	1910.15
	**EA_0.01**	156.35	384.78	1.00	-1190.24	1502.95
	**EA_0.1**	283.97	384.78	1.00	-1062.62	1630.57
	**EA_1**	3450.94	384.78	0.00	2104.35	4797.54
	**EA_10**	4008.09	314.17	0.00	2908.60	5107.58
	**PTZ**	-194.13	314.17	1.00	-1293.62	905.36
	**VPA**	2574.94	314.17	0.00	1475.45	3674.43
**E100_1**	**E25_0.01**	-16.82	384.78	1.00	-1363.41	1329.78
	**E25_0.1**	-419.82	384.78	1.00	-1766.41	926.78
	**E25_1**	-131.12	384.78	1.00	-1477.71	1215.48
	**E25_10**	3331.22	384.78	0.00	1984.63	4677.82
	**E100_0.01**	-388.27	384.78	1.00	-1734.87	958.33
	**E100_0.1**	-587.54	384.78	0.99	-1934.13	759.06
	**E100_1**	4498.13	384.78	0.00	3151.53	5844.72
	**E100_10**	-23.98	384.78	1.00	-1370.57	1322.62
	**EA_0.01**	-431.18	384.78	1.00	-1777.78	915.41
	**EA_0.1**	-303.56	384.78	1.00	-1650.16	1043.03
	**EA_1**	2863.41	384.78	0.00	1516.81	4210.00
	**EA_10**	3420.56	314.17	0.00	2321.07	4520.05
	**PTZ**	-781.66	314.17	0.54	-1881.15	317.83
	**VPA**	1987.40	314.17	0.00	887.91	3086.89
**E100_10**	**E25_0.01**	-4514.95	384.78	0.00	-5861.54	-3168.35
	**E25_0.1**	-4917.95	384.78	0.00	-6264.54	-3571.35
	**E25_1**	-4629.25	384.78	0.00	-5975.84	-3282.65

	**E25_10**	-1166.91	384.78	0.19	-2513.50	179.69
	**E100_0.01**	-4886.40	384.78	0.00	-6232.99	-3539.80
	**E100_0.1**	-5085.66	384.78	0.00	-6432.26	-3739.07
	**E100_1**	-4498.13	384.78	0.00	-5844.72	-3151.53
	**E100_10**	-4522.11	384.78	0.00	-5868.70	-3175.51
	**EA_0.01**	-4929.31	384.78	0.00	-6275.91	-3582.72
	**EA_0.1**	-4801.69	384.78	0.00	-6148.29	-3455.10
	**EA_1**	-1634.72	384.78	0.00	-2981.32	-288.13
	**EA_10**	-1077.57	314.17	0.06	-2177.06	21.92
	**PTZ**	-5279.79	314.17	0.00	-6379.28	-4180.30
	**VPA**	-2510.73	314.17	0.00	-3610.22	-1411.24
**EA_0.01**	**E25_0.01**	7.16	384.78	1.00	-1339.44	1353.75
	**E25_0.1**	-395.84	384.78	1.00	-1742.44	950.75
	**E25_1**	-107.14	384.78	1.00	-1453.74	1239.45
	**E25_10**	3355.20	384.78	0.00	2008.61	4701.80
	**E100_0.01**	-364.29	384.78	1.00	-1710.89	982.30
	**E100_0.1**	-563.56	384.78	0.99	-1910.15	783.04
	**E100_1**	23.98	384.78	1.00	-1322.62	1370.57
	**E100_10**	4522.11	384.78	0.00	3175.51	5868.70
	**EA_0.01**	-407.20	384.78	1.00	-1753.80	939.39
	**EA_0.1**	-279.59	384.78	1.00	-1626.18	1067.01
	**EA_1**	2887.39	384.78	0.00	1540.79	4233.98
	**EA_10**	3444.54	314.17	0.00	2345.05	4544.03
	**PTZ**	-757.68	314.17	0.60	-1857.18	341.81
	**VPA**	2011.38	314.17	0.00	911.89	3110.87
**EA_0.1**	**E25_0.01**	414.36	384.78	1.00	-932.23	1760.96
	**E25_0.1**	11.36	384.78	1.00	-1335.23	1357.96
	**E25_1**	300.06	384.78	1.00	-1046.53	1646.66
	**E25_10**	3762.41	384.78	0.00	2415.81	5109.00
	**E100_0.01**	42.91	384.78	1.00	-1303.68	1389.51
	**E100_0.1**	-156.35	384.78	1.00	-1502.95	1190.24
	**E100_1**	431.18	384.78	1.00	-915.41	1777.78
	**E100_10**	4929.31	384.78	0.00	3582.72	6275.91
	**EA_0.01**	407.20	384.78	1.00	-939.39	1753.80
	**EA_0.1**	127.62	384.78	1.00	-1218.98	1474.21
	**EA_1**	3294.59	384.78	0.00	1948.00	4641.19
	**EA_10**	3851.74	314.17	0.00	2752.25	4951.23
	**PTZ**	-350.48	314.17	1.00	-1449.97	749.01
	**VPA**	2418.59	314.17	0.00	1319.09	3518.08
**EA_1**	**E25_0.01**	286.74	384.78	1.00	-1059.85	1633.34
	**E25_0.1**	-116.26	384.78	1.00	-1462.85	1230.34
	**E25_1**	172.44	384.78	1.00	-1174.15	1519.04
	**E25_10**	3634.79	384.78	0.00	2288.19	4981.38
	**E100_0.01**	-84.71	384.78	1.00	-1431.30	1261.89
	**E100_0.1**	-283.97	384.78	1.00	-1630.57	1062.62
	**E100_1**	303.56	384.78	1.00	-1043.03	1650.16
	**E100_10**	4801.69	384.78	0.00	3455.10	6148.29
	**EA_0.01**	279.59	384.78	1.00	-1067.01	1626.18

	**EA_0.1**	-127.62	384.78	1.00	-1474.21	1218.98
	**EA_1**	3166.97	384.78	0.00	1820.38	4513.57
	**EA_10**	3724.12	314.17	0.00	2624.63	4823.61
	**PTZ**	-478.10	314.17	0.99	-1577.59	621.39
	**VPA**	2290.97	314.17	0.00	1191.47	3390.46
**EA_10**	**E25_0.01**	-2880.23	384.78	0.00	-4226.82	-1533.63
	**E25_0.1**	-3283.23	384.78	0.00	-4629.82	-1936.63
	**E25_1**	-2994.53	384.78	0.00	-4341.12	-1647.93
	**E25_10**	467.82	384.78	1.00	-878.78	1814.41
	**E100_0.01**	-3251.68	384.78	0.00	-4598.27	-1905.08
	**E100_0.1**	-3450.94	384.78	0.00	-4797.54	-2104.35
	**E100_1**	-2863.41	384.78	0.00	-4210.00	-1516.81
	**E100_10**	1634.72	384.78	0.00	288.13	2981.32
	**EA_0.01**	-2887.39	384.78	0.00	-4233.98	-1540.79
	**EA_0.1**	-3294.59	384.78	0.00	-4641.19	-1948.00
	**EA_1**	-3166.97	384.78	0.00	-4513.57	-1820.38
	**EA_10**	557.15	314.17	0.95	-542.34	1656.64
	**PTZ**	-3645.07	314.17	0.00	-4744.56	-2545.58
	**VPA**	-876.01	314.17	0.32	-1975.50	223.49
**DMSO**	**E25_0.01**	-3437.38	314.17	0.00	-4536.87	-2337.89
	**E25_0.1**	-3840.38	314.17	0.00	-4939.87	-2740.89
	**E25_1**	-3551.68	314.17	0.00	-4651.17	-2452.19
	**E25_10**	-89.33	314.17	1.00	-1188.82	1010.16
	**E100_0.01**	-3808.83	314.17	0.00	-4908.32	-2709.34
	**E100_0.1**	-4008.09	314.17	0.00	-5107.58	-2908.60
	**E100_1**	-3420.56	314.17	0.00	-4520.05	-2321.07
	**E100_10**	1077.57	314.17	0.06	-21.92	2177.06
	**EA_0.01**	-3444.54	314.17	0.00	-4544.03	-2345.05
	**EA_0.1**	-3851.74	314.17	0.00	-4951.23	-2752.25
	**EA_1**	-3724.12	314.17	0.00	-4823.61	-2624.63
	**EA_10**	-557.15	314.17	0.95	-1656.64	542.34
	**PTZ**	-4202.22	222.15	0.00	-4979.68	-3424.76
	**VPA**	-1433.16	222.15	0.00	-2210.61	-655.70
**PTZ**	**E25_0.01**	764.84	314.17	0.58	-334.65	1864.33
	**E25_0.1**	361.84	314.17	1.00	-737.65	1461.33
	**E25_1**	650.54	314.17	0.83	-448.95	1750.03
	**E25_10**	4112.89	314.17	0.00	3013.40	5212.38
	**E100_0.01**	393.39	314.17	1.00	-706.10	1492.88
	**E100_0.1**	194.13	314.17	1.00	-905.36	1293.62
	**E100_1**	781.66	314.17	0.54	-317.83	1881.15
	**E100_10**	5279.79	314.17	0.00	4180.30	6379.28
	**EA_0.01**	757.68	314.17	0.60	-341.81	1857.18
	**EA_0.1**	350.48	314.17	1.00	-749.01	1449.97
	**EA_1**	478.10	314.17	0.99	-621.39	1577.59
	**EA_10**	3645.07	314.17	0.00	2545.58	4744.56
	**DMSO**	4202.22	222.15	0.00	3424.76	4979.68
	**VPA**	2769.07	222.15	0.00	1991.61	3546.52
**VPA**	**E25_0.01**	-2004.22	314.17	0.00	-3103.71	-904.73

	**E25_0.1**	-2407.22	314.17	0.00	-3506.71	-1307.73
	**E25_1**	-2118.52	314.17	0.00	-3218.01	-1019.03
	**E25_10**	1343.82	314.17	0.00	244.33	2443.31
	**E100_0.01**	-2375.67	314.17	0.00	-3475.16	-1276.18
	**E100_0.1**	-2574.94	314.17	0.00	-3674.43	-1475.45
	**E100_1**	-1987.40	314.17	0.00	-3086.89	-887.91
	**E100_10**	2510.73	314.17	0.00	1411.24	3610.22
	**EA_0.01**	-2011.38	314.17	0.00	-3110.87	-911.89
	**EA_0.1**	-2418.59	314.17	0.00	-3518.08	-1319.09
	**EA_1**	-2290.97	314.17	0.00	-3390.46	-1191.47
	**EA_10**	876.01	314.17	0.32	-223.49	1975.50
	**DMSO**	1433.16	222.15	0.00	655.70	2210.61
	**PTZ**	-2769.07	222.15	0.00	-3546.52	-1991.61
**CAN-3**						
**E25_0.01**	**E25_0.01**	860.75	458.56	0.92	-744.03	2465.53
	**E25_0.1**	736.48	458.56	0.98	-868.30	2341.26
	**E25_1**	4863.95	458.56	0.00	3259.17	6468.73
	**E25_10**	5.19	458.56	1.00	-1599.59	1609.97
	**E100_0.01**	1500.76	458.56	0.10	-104.02	3105.54
	**E100_0.1**	1829.03	458.56	0.01	224.25	3433.81
	**E100_1**	7073.75	458.56	0.00	5468.97	8678.53
	**E100_10**	424.60	458.56	1.00	-1180.18	2029.38
	**EA_0.01**	91.89	458.56	1.00	-1512.89	1696.67
	**EA_0.1**	203.79	458.56	1.00	-1400.99	1808.57
	**EA_1**	4400.41	458.56	0.00	2795.63	6005.19
	**EA_10**	5218.54	374.41	0.00	3908.24	6528.84
	**PTZ**	506.90	374.41	1.00	-803.40	1817.20
	**VPA**	3260.31	374.41	0.00	1950.01	4570.61
**E25_0.1**	**E25_0.01**	-860.75	458.56	0.92	-2465.53	744.03
	**E25_0.1**	-124.27	458.56	1.00	-1729.06	1480.51
	**E25_1**	4003.20	458.56	0.00	2398.42	5607.98
	**E25_10**	-855.56	458.56	0.92	-2460.34	749.22
	**E100_0.01**	640.01	458.56	1.00	-964.77	2244.79
	**E100_0.1**	968.28	458.56	0.81	-636.50	2573.06
	**E100_1**	6212.99	458.56	0.00	4608.21	7817.78
	**E100_10**	-436.15	458.56	1.00	-2040.94	1168.63
	**EA_0.01**	-768.86	458.56	0.97	-2373.64	835.92
	**EA_0.1**	-656.96	458.56	0.99	-2261.75	947.82
	**EA_1**	3539.66	458.56	0.00	1934.88	5144.44
	**EA_10**	4357.79	374.41	0.00	3047.49	5668.08
	**PTZ**	-353.85	374.41	1.00	-1664.15	956.45
	**VPA**	2399.56	374.41	0.00	1089.26	3709.86
**E25_1**	**E25_0.01**	-736.48	458.56	0.98	-2341.26	868.30
	**E25_0.1**	124.27	458.56	1.00	-1480.51	1729.06
	**E25_1**	4127.47	458.56	0.00	2522.69	5732.26
	**E25_10**	-731.29	458.56	0.98	-2336.07	873.49
	**E100_0.01**	764.29	458.56	0.97	-840.50	2369.07
	**E100_0.1**	1092.55	458.56	0.62	-512.23	2697.33

	**E100_1**	6337.27	458.56	0.00	4732.49	7942.05
	**E100_10**	-311.88	458.56	1.00	-1916.66	1292.90
	**EA_0.01**	-644.58	458.56	1.00	-2249.36	960.20
	**EA_0.1**	-532.69	458.56	1.00	-2137.47	1072.09
	**EA_1**	3663.93	458.56	0.00	2059.15	5268.71
	**EA_10**	4482.06	374.41	0.00	3171.76	5792.36
	**PTZ**	-229.58	374.41	1.00	-1539.87	1080.72
	**VPA**	2523.84	374.41	0.00	1213.54	3834.13
**E25_10**	**E25_0.01**	-4863.95	458.56	0.00	-6468.73	-3259.17
	**E25_0.1**	-4003.20	458.56	0.00	-5607.98	-2398.42
	**E25_1**	-4127.47	458.56	0.00	-5732.26	-2522.69
	**E25_10**	-4858.76	458.56	0.00	-6463.54	-3253.98
	**E100_0.01**	-3363.19	458.56	0.00	-4967.97	-1758.41
	**E100_0.1**	-3034.92	458.56	0.00	-4639.70	-1430.14
	**E100_1**	2209.79	458.56	0.00	605.01	3814.58
	**E100_10**	-4439.35	458.56	0.00	-6044.14	-2834.57
	**EA_0.01**	-4772.06	458.56	0.00	-6376.84	-3167.28
	**EA_0.1**	-4660.17	458.56	0.00	-6264.95	-3055.38
	**EA_1**	-463.54	458.56	1.00	-2068.32	1141.24
	**EA_10**	354.59	374.41	1.00	-955.71	1664.88
	**PTZ**	-4357.05	374.41	0.00	-5667.35	-3046.75
	**VPA**	-1603.64	374.41	0.00	-2913.94	-293.34
**E100_0.01**	**E25_0.01**	-5.19	458.56	1.00	-1609.97	1599.59
	**E25_0.1**	855.56	458.56	0.92	-749.22	2460.34
	**E25_1**	731.29	458.56	0.98	-873.49	2336.07
	**E25_10**	4858.76	458.56	0.00	3253.98	6463.54
	**E100_0.01**	1495.57	458.56	0.10	-109.21	3100.35
	**E100_0.1**	1823.84	458.56	0.01	219.06	3428.62
	**E100_1**	7068.56	458.56	0.00	5463.78	8673.34
	**E100_10**	419.41	458.56	1.00	-1185.37	2024.19
	**EA_0.01**	86.70	458.56	1.00	-1518.08	1691.48
	**EA_0.1**	198.60	458.56	1.00	-1406.18	1803.38
	**EA_1**	4395.22	458.56	0.00	2790.44	6000.00
	**EA_10**	5213.35	374.41	0.00	3903.05	6523.64
	**PTZ**	501.71	374.41	1.00	-808.59	1812.01
	**VPA**	3255.12	374.41	0.00	1944.82	4565.42
**E100_0.1**	**E25_0.01**	-1500.76	458.56	0.10	-3105.54	104.02
	**E25_0.1**	-640.01	458.56	1.00	-2244.79	964.77
	**E25_1**	-764.29	458.56	0.97	-2369.07	840.50
	**E25_10**	3363.19	458.56	0.00	1758.41	4967.97
	**E100_0.01**	-1495.57	458.56	0.10	-3100.35	109.21
	**E100_0.1**	328.27	458.56	1.00	-1276.51	1933.05
	**E100_1**	5572.98	458.56	0.00	3968.20	7177.76
	**E100_10**	-1076.17	458.56	0.65	-2680.95	528.62
	**EA_0.01**	-1408.87	458.56	0.17	-3013.65	195.91
	**EA_0.1**	-1296.98	458.56	0.30	-2901.76	307.80
	**EA_1**	2899.65	458.56	0.00	1294.87	4504.43
	**EA_10**	3717.77	374.41	0.00	2407.48	5028.07

	**PTZ**	-993.86	374.41	0.41	-2304.16	316.44
	**VPA**	1759.55	374.41	0.00	449.25	3069.85
**E100_1**	**E25_0.01**	-1829.03	458.56	0.01	-3433.81	-224.25
	**E25_0.1**	-968.28	458.56	0.81	-2573.06	636.50
	**E25_1**	-1092.55	458.56	0.62	-2697.33	512.23
	**E25_10**	3034.92	458.56	0.00	1430.14	4639.70
	**E100_0.01**	-1823.84	458.56	0.01	-3428.62	-219.06
	**E100_0.1**	-328.27	458.56	1.00	-1933.05	1276.51
	**E100_1**	5244.72	458.56	0.00	3639.93	6849.50
	**E100_10**	-1404.43	458.56	0.17	-3009.21	200.35
	**EA_0.01**	-1737.14	458.56	0.02	-3341.92	-132.36
	**EA_0.1**	-1625.24	458.56	0.04	-3230.02	-20.46
	**EA_1**	2571.38	458.56	0.00	966.60	4176.16
	**EA_10**	3389.51	374.41	0.00	2079.21	4699.80
	**PTZ**	-1322.13	374.41	0.05	-2632.43	-11.83
	**VPA**	1431.28	374.41	0.02	120.98	2741.58
**E100_10**	**E25_0.01**	-7073.75	458.56	0.00	-8678.53	-5468.97
	**E25_0.1**	-6212.99	458.56	0.00	-7817.78	-4608.21
	**E25_1**	-6337.27	458.56	0.00	-7942.05	-4732.49
	**E25_10**	-2209.79	458.56	0.00	-3814.58	-605.01
	**E100_0.01**	-7068.56	458.56	0.00	-8673.34	-5463.78
	**E100_0.1**	-5572.98	458.56	0.00	-7177.76	-3968.20
	**E100_1**	-5244.72	458.56	0.00	-6849.50	-3639.93
	**E100_10**	-6649.15	458.56	0.00	-8253.93	-5044.37
	**EA_0.01**	-6981.85	458.56	0.00	-8586.63	-5377.07
	**EA_0.1**	-6869.96	458.56	0.00	-8474.74	-5265.18
	**EA_1**	-2673.34	458.56	0.00	-4278.12	-1068.56
	**EA_10**	-1855.21	374.41	0.00	-3165.51	-544.91
	**PTZ**	-6566.84	374.41	0.00	-7877.14	-5256.55
	**VPA**	-3813.43	374.41	0.00	-5123.73	-2503.14
**EA_0.01**	**E25_0.01**	-424.60	458.56	1.00	-2029.38	1180.18
	**E25_0.1**	436.15	458.56	1.00	-1168.63	2040.94
	**E25_1**	311.88	458.56	1.00	-1292.90	1916.66
	**E25_10**	4439.35	458.56	0.00	2834.57	6044.14
	**E100_0.01**	-419.41	458.56	1.00	-2024.19	1185.37
	**E100_0.1**	1076.17	458.56	0.65	-528.62	2680.95
	**E100_1**	1404.43	458.56	0.17	-200.35	3009.21
	**E100_10**	6649.15	458.56	0.00	5044.37	8253.93
	**EA_0.01**	-332.70	458.56	1.00	-1937.48	1272.08
	**EA_0.1**	-220.81	458.56	1.00	-1825.59	1383.97
	**EA_1**	3975.81	458.56	0.00	2371.03	5580.59
	**EA_10**	4793.94	374.41	0.00	3483.64	6104.24
	**PTZ**	82.30	374.41	1.00	-1227.99	1392.60
	**VPA**	2835.72	374.41	0.00	1525.42	4146.01
**EA_0.1**	**E25_0.01**	-91.89	458.56	1.00	-1696.67	1512.89
	**E25_0.1**	768.86	458.56	0.97	-835.92	2373.64
	**E25_1**	644.58	458.56	1.00	-960.20	2249.36
	**E25_10**	4772.06	458.56	0.00	3167.28	6376.84

	**E100_0.01**	-86.70	458.56	1.00	-1691.48	1518.08
	**E100_0.1**	1408.87	458.56	0.17	-195.91	3013.65
	**E100_1**	1737.14	458.56	0.02	132.36	3341.92
	**E100_10**	6981.85	458.56	0.00	5377.07	8586.63
	**EA_0.01**	332.70	458.56	1.00	-1272.08	1937.48
	**EA_0.1**	111.89	458.56	1.00	-1492.89	1716.67
	**EA_1**	4308.52	458.56	0.00	2703.74	5913.30
	**EA_10**	5126.64	374.41	0.00	3816.35	6436.94
	**PTZ**	415.01	374.41	1.00	-895.29	1725.31
	**VPA**	3168.42	374.41	0.00	1858.12	4478.72
**EA_1**	**E25_0.01**	-203.79	458.56	1.00	-1808.57	1400.99
	**E25_0.1**	656.96	458.56	0.99	-947.82	2261.75
	**E25_1**	532.69	458.56	1.00	-1072.09	2137.47
	**E25_10**	4660.17	458.56	0.00	3055.38	6264.95
	**E100_0.01**	-198.60	458.56	1.00	-1803.38	1406.18
	**E100_0.1**	1296.98	458.56	0.30	-307.80	2901.76
	**E100_1**	1625.24	458.56	0.04	20.46	3230.02
	**E100_10**	6869.96	458.56	0.00	5265.18	8474.74
	**EA_0.01**	220.81	458.56	1.00	-1383.97	1825.59
	**EA_0.1**	-111.89	458.56	1.00	-1716.67	1492.89
	**EA_1**	4196.62	458.56	0.00	2591.84	5801.40
	**EA_10**	5014.75	374.41	0.00	3704.45	6325.05
	**PTZ**	303.12	374.41	1.00	-1007.18	1613.41
	**VPA**	3056.53	374.41	0.00	1746.23	4366.82
**EA_10**	**E25_0.01**	-4400.41	458.56	0.00	-6005.19	-2795.63
	**E25_0.1**	-3539.66	458.56	0.00	-5144.44	-1934.88
	**E25_1**	-3663.93	458.56	0.00	-5268.71	-2059.15
	**E25_10**	463.54	458.56	1.00	-1141.24	2068.32
	**E100_0.01**	-4395.22	458.56	0.00	-6000.00	-2790.44
	**E100_0.1**	-2899.65	458.56	0.00	-4504.43	-1294.87
	**E100_1**	-2571.38	458.56	0.00	-4176.16	-966.60
	**E100_10**	2673.34	458.56	0.00	1068.56	4278.12
	**EA_0.01**	-3975.81	458.56	0.00	-5580.59	-2371.03
	**EA_0.1**	-4308.52	458.56	0.00	-5913.30	-2703.74
	**EA_1**	-4196.62	458.56	0.00	-5801.40	-2591.84
	**EA_10**	818.13	374.41	0.76	-492.17	2128.43
	**PTZ**	-3893.51	374.41	0.00	-5203.81	-2583.21
	**VPA**	-1140.10	374.41	0.18	-2450.40	170.20
**DMSO**	**E25_0.01**	-5218.54	374.41	0.00	-6528.84	-3908.24
	**E25_0.1**	-4357.79	374.41	0.00	-5668.08	-3047.49
	**E25_1**	-4482.06	374.41	0.00	-5792.36	-3171.76
	**E25_10**	-354.59	374.41	1.00	-1664.88	955.71
	**E100_0.01**	-5213.35	374.41	0.00	-6523.64	-3903.05
	**E100_0.1**	-3717.77	374.41	0.00	-5028.07	-2407.48
	**E100_1**	-3389.51	374.41	0.00	-4699.80	-2079.21
	**E100_10**	1855.21	374.41	0.00	544.91	3165.51
	**EA_0.01**	-4793.94	374.41	0.00	-6104.24	-3483.64
	**EA_0.1**	-5126.64	374.41	0.00	-6436.94	-3816.35

	**EA_1**	-5014.75	374.41	0.00	-6325.05	-3704.45
	**EA_10**	-818.13	374.41	0.76	-2128.43	492.17
	**PTZ**	-4711.64	264.75	0.00	-5638.16	-3785.11
	**VPA**	-1958.22	264.75	0.00	-2884.75	-1031.70
**PTZ**	**E25_0.01**	-506.90	374.41	1.00	-1817.20	803.40
	**E25_0.1**	353.85	374.41	1.00	-956.45	1664.15
	**E25_1**	229.58	374.41	1.00	-1080.72	1539.87
	**E25_10**	4357.05	374.41	0.00	3046.75	5667.35
	**E100_0.01**	-501.71	374.41	1.00	-1812.01	808.59
	**E100_0.1**	993.86	374.41	0.41	-316.44	2304.16
	**E100_1**	1322.13	374.41	0.05	11.83	2632.43
	**E100_10**	6566.84	374.41	0.00	5256.55	7877.14
	**EA_0.01**	-82.30	374.41	1.00	-1392.60	1227.99
	**EA_0.1**	-415.01	374.41	1.00	-1725.31	895.29
	**EA_1**	-303.12	374.41	1.00	-1613.41	1007.18
	**EA_10**	3893.51	374.41	0.00	2583.21	5203.81
	**DMSO**	4711.64	264.75	0.00	3785.11	5638.16
	**VPA**	2753.41	264.75	0.00	1826.89	3679.93
**VPA**	**E25_0.01**	-3260.31	374.41	0.00	-4570.61	-1950.01
	**E25_0.1**	-2399.56	374.41	0.00	-3709.86	-1089.26
	**E25_1**	-2523.84	374.41	0.00	-3834.13	-1213.54
	**E25_10**	1603.64	374.41	0.00	293.34	2913.94
	**E100_0.01**	-3255.12	374.41	0.00	-4565.42	-1944.82
	**E100_0.1**	-1759.55	374.41	0.00	-3069.85	-449.25
	**E100_1**	-1431.28	374.41	0.02	-2741.58	-120.98
	**E100_10**	3813.43	374.41	0.00	2503.14	5123.73
	**EA_0.01**	-2835.72	374.41	0.00	-4146.01	-1525.42
	**EA_0.1**	-3168.42	374.41	0.00	-4478.72	-1858.12
	**EA_1**	-3056.53	374.41	0.00	-4366.82	-1746.23
	**EA_10**	1140.10	374.41	0.18	-170.20	2450.40
	**DMSO**	1958.22	264.75	0.00	1031.70	2884.75
	**PTZ**	-2753.41	264.75	0.00	-3679.93	-1826.89

Phase 3

Table S16. Effect Sizes

		Point Estimate	95% Confidence Interval Lower	Upper
**CAN-1**	**Eta-squared**	0.23	0.10	0.32
	**Epsilon-squared**	0.21	0.07	0.30
	**Omega-squared Fixed-effect**	0.20	0.07	0.30
	**Omega-squared Random-effect**	0.05	0.01	0.08
**CAN-2**	**Eta-squared**	0.30	0.16	0.39
	**Epsilon-squared**	0.28	0.13	0.37
	**Omega-squared Fixed-effect**	0.28	0.13	0.37
	**Omega-squared Random-effect**	0.07	0.03	0.10
**CAN-3**	**Eta-squared**	0.33	0.19	0.42
	**Epsilon-squared**	0.31	0.16	0.40
	**Omega-squared Fixed-effect**	0.30	0.16	0.40
	**Omega-squared Random-effect**	0.08	0.04	0.12

Table S17. Tukey HSD Post-hoc Tests

	Test solution (µg/ml)	Test solution (µg/ml)	Mean Difference	Std. Error	Sig.	95% Confidence Interval	
						Lower Bound	Upper Bound
**CAN-1**	**E25 10**	**E100 10**	-638.83	375.36	0.53	-1723.70	446.03
		**EA 10**	-1495.77028*	375.36	0.00	-2580.64	-410.90
		**CBD 12**	-2842.54816*	530.85	0.00	-4376.78	-1308.32
		**CBD 15**	-1744.09992*	530.85	0.02	-3278.33	-209.87
		**CBD 18**	-55.82	530.85	1.00	-1590.05	1478.42
	**E100 10**	**E25 10**	638.83	375.36	0.53	-446.03	1723.70
		**EA 10**	-856.94	375.36	0.21	-1941.80	227.93
		**CBD 12**	-2203.71343*	530.85	0.00	-3737.95	-669.48
		**CBD 15**	-1105.27	530.85	0.30	-2639.50	428.97
		**CBD 18**	583.02	530.85	0.88	-951.21	2117.25
	**EA 10**	**E25 10**	1495.77028*	375.36	0.00	410.90	2580.64
		**E100 10**	856.94	375.36	0.21	-227.93	1941.80
		**CBD 12**	-1346.78	530.85	0.12	-2881.01	187.46
		**CBD 15**	-248.33	530.85	1.00	-1782.56	1285.90
		**CBD 18**	1439.95	530.85	0.08	-94.28	2974.19
	**CBD 12**	**E25 10**	2842.54816*	530.85	0.00	1308.32	4376.78
		**E100 10**	2203.71343*	530.85	0.00	669.48	3737.95
		**EA 10**	1346.78	530.85	0.12	-187.46	2881.01
		**CBD 15**	1098.45	650.15	0.54	-780.60	2977.49
		**CBD 18**	2786.73146*	650.15	0.00	907.69	4665.78
	**CBD 15**	**E25 10**	1744.09992*	530.85	0.02	209.87	3278.33
		**E100 10**	1105.27	530.85	0.30	-428.97	2639.50
		**EA 10**	248.33	530.85	1.00	-1285.90	1782.56
		**CBD 12**	-1098.45	650.15	0.54	-2977.49	780.60
		**CBD 18**	1688.28	650.15	0.10	-190.76	3567.33

	**CBD 18**	**E25 10**	55.82	530.85	1.00	-1478.42	1590.05
		**E100 10**	-583.02	530.85	0.88	-2117.25	951.21
		**EA 10**	-1439.95	530.85	0.08	-2974.19	94.28
		**CBD 12**	-2786.73146*	650.15	0.00	-4665.78	-907.69
		**CBD 15**	-1688.28	650.15	0.10	-3567.33	190.76
**CAN-2**	**E25 10**	**E100 10**	1166.90556*	307.69	0.00	277.64	2056.17
		**EA 10**	-467.82	307.69	0.65	-1357.08	421.45
		**CBD 12**	-1418.03899*	435.13	0.02	-2675.65	-160.43
		**CBD 15**	-319.59	435.13	0.98	-1577.20	938.02
		**CBD 18**	1368.69247*	435.13	0.02	111.08	2626.30
	**E100 10**	**E25 10**	-1166.90556*	307.69	0.00	-2056.17	-277.64
		**EA 10**	-1634.72139*	307.69	0.00	-2523.98	-745.46
		**CBD 12**	-2584.94454*	435.13	0.00	-3842.55	-1327.34
		**CBD 15**	-1486.49631*	435.13	0.01	-2744.10	-228.89
		**CBD 18**	201.79	435.13	1.00	-1055.82	1459.40
	**EA 10**	**E25 10**	467.82	307.69	0.65	-421.45	1357.08
		**E100 10**	1634.72139*	307.69	0.00	745.46	2523.98
		**CBD 12**	-950.22	435.13	0.25	-2207.83	307.38
		**CBD 15**	148.23	435.13	1.00	-1109.38	1405.83
		**CBD 18**	1836.50830*	435.13	0.00	578.90	3094.12
	**CBD 12**	**E25 10**	1418.03899*	435.13	0.02	160.43	2675.65
		**E100 10**	2584.94454*	435.13	0.00	1327.34	3842.55
		**EA 10**	950.22	435.13	0.25	-307.38	2207.83
		**CBD 15**	1098.45	532.93	0.31	-441.80	2638.70
		**CBD 18**	2786.73146*	532.93	0.00	1246.48	4326.98
	**CBD 15**	**E25 10**	319.59	435.13	0.98	-938.02	1577.20
		**E100 10**	1486.49631*	435.13	0.01	228.89	2744.10
		**EA 10**	-148.23	435.13	1.00	-1405.83	1109.38
		**CBD 12**	-1098.45	532.93	0.31	-2638.70	441.80
		**CBD 18**	1688.28322*	532.93	0.02	148.03	3228.53
	**CBD 18**	**E25 10**	-1368.69247*	435.13	0.02	-2626.30	-111.08
		**E100 10**	-201.79	435.13	1.00	-1459.40	1055.82
		**EA 10**	-1836.50830*	435.13	0.00	-3094.12	-578.90
		**CBD 12**	-2786.73146*	532.93	0.00	-4326.98	-1246.48
		**CBD 15**	-1688.28322*	532.93	0.02	-3228.53	-148.03
**CAN-3**	**E25 10**	**E100 10**	2209.79444*	400.21	0.00	1053.11	3366.48
		**EA 10**	-463.54	400.21	0.86	-1620.22	693.14
		**CBD 12**	-934.93	565.99	0.57	-2570.72	700.87
		**CBD 15**	163.52	565.99	1.00	-1472.27	1799.32
		**CBD 18**	1851.80580*	565.99	0.02	216.01	3487.60
	**E100 10**	**E25 10**	-2209.79444*	400.21	0.00	-3366.48	-1053.11
		**EA 10**	-2673.33667*	400.21	0.00	-3830.02	-1516.66
		**CBD 12**	-3144.72010*	565.99	0.00	-4780.51	-1508.93
		**CBD 15**	-2046.27187*	565.99	0.01	-3682.07	-410.48
		**CBD 18**	-357.99	565.99	0.99	-1993.78	1277.80
	**EA 10**	**E25 10**	463.54	400.21	0.86	-693.14	1620.22
		**E100 10**	2673.33667*	400.21	0.00	1516.66	3830.02
		**CBD 12**	-471.38	565.99	0.96	-2107.18	1164.41

	**CBD 15**	627.06	565.99	0.88	-1008.73	2262.86
	**CBD 18**	2315.34802*	565.99	0.00	679.55	3951.14
**CBD 12**	**E25 10**	934.93	565.99	0.57	-700.87	2570.72
	**E100 10**	3144.72010*	565.99	0.00	1508.93	4780.51
	**EA 10**	471.38	565.99	0.96	-1164.41	2107.18
	**CBD 15**	1098.45	693.19	0.61	-904.98	3101.88
	**CBD 18**	2786.73146*	693.19	0.00	783.30	4790.16
**CBD 15**	**E25 10**	-163.52	565.99	1.00	-1799.32	1472.27
	**E100 10**	2046.27187*	565.99	0.01	410.48	3682.07
	**EA 10**	-627.06	565.99	0.88	-2262.86	1008.73
	**CBD 12**	-1098.45	693.19	0.61	-3101.88	904.98
	**CBD 18**	1688.28	693.19	0.15	-315.15	3691.71
**CBD 18**	**E25 10**	-1851.80580*	565.99	0.02	-3487.60	-216.01
	**E100 10**	357.99	565.99	0.99	-1277.80	1993.78
	**EA 10**	-2315.34802*	565.99	0.00	-3951.14	-679.55
	**CBD 12**	-2786.73146*	693.19	0.00	-4790.16	-783.30
	**CBD 15**	-1688.28	693.19	0.15	-3691.71	315.15
